# Dynamic Synthesis of Multi‐Modal Representations for CITE‐seq Data Integration and Analysis

**DOI:** 10.1002/advs.202509247

**Published:** 2025-09-08

**Authors:** Yinan Shi, Yanchi Su, Yue Cheng, Ka‐Chun Wong, Yunhe Wang, Xiangtao Li

**Affiliations:** ^1^ School of Artificial Intelligence Jilin University Changchun 130012 China; ^2^ School of Information Science and Technology Northeast Normal University Changchun 130024 China; ^3^ Department of Computer Science City University of Hong Kong Hong Kong SAR 999077 China; ^4^ School of Artificial Intelligence Hebei University of Technology Tianjin 300401 China

**Keywords:** autoencoder, clustering, deep learning, multi‐omics

## Abstract

Single‐cell multi‐omics technologies are pivotal for deciphering the complexities of biological systems, with Cellular Indexing of Transcriptomes and Epitopes by Sequencing (CITE‐seq) emerging as a particularly valuable approach. The dual‐modality capability makes CITE‐seq particularly advantageous for dissecting cellular heterogeneity and understanding the dynamic interplay between transcriptomic and proteomic landscapes. However, existing computational models for integrating these two modalities often struggle to capture the complex, non‐linear interactions between RNA and antibody‐derived tags (ADTs), and are computationally intensive. To address these issues, scMHVA, a novel and lightweight framework designed to integrate the diverse modalities of CITE‐seq data, is proposed. scMHVA utilizes an adaptive dynamic synthesis module to generate consolidated yet heterogeneous embeddings from RNA and ADT modalities. Subsequently, scMHVA enhances inter‐modality correlations within the joint representation by applying a multi‐head self‐attention mechanism, effectively capturing the intricate mapping relationships between mRNA expression levels and protein abundance. Extensive experiments demonstrate that scMHVA consistently outperformed existing single‐modal and multi‐modal clustering methods across CITE‐seq datasets of varying scales, exhibiting linear runtime scalability and effectively eliminating batch effects, thereby establishing it as a robust tool for large‐scale CITE‐seq data analysis. Additionally, it is demonstrated that scMHVA successfully annotates different cell types in a published mouse thymocyte dataset and reveals dynamics of immune cell development.

## Graphical abstract

The accurate integration of CITE‐seq modalities faces challenges due to complex RNA‐protein nonlinear interactions and computational inefficiencies. A lightweight framework that adaptively fuses RNA and ADT modalities, and refines them through fine‐grained learning is developed to address this issue. This model enhances multi‐modal integration and clustering performance, revealing dynamics of immune cell development in mouse thymocytes.

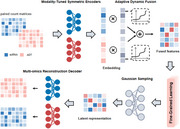

## 1 Introduction

Single‐cell multi‐omics (scMulti‐omics) technologies enable simultaneous profiling of multiple molecular features within individual cells, including gene expression, protein abundance, and chromatin accessibility.^[^ ^1^, ^2^ ^]^ Among these approaches, CITE‐seq^[^ ^3^ ^]^ offers the unique ability to integrate transcriptomic data (capturing gene expression) with epitope‐level protein data (reflecting cell‐surface markers and signaling states) in the same cells. This combined modality provides a more holistic view of cellular phenotypes, enabling more accurate cell clustering, which is a foundational step for downstream analyses such as cell type annotation, trajectory inference, and immune cell state characterization. In practice, accurate clustering of CITE‐seq data facilitates more precise cell type identification,^[^ ^4^ ^]^ helps unveil the intricate interplay among diverse cell types within complex tissues,^[^ ^5^ ^]^ and reveals biological insights that are not discernible using a single data modality alone,^[^ ^6^ ^]^ including identifying rare subcellular organelles and subcellular feature inference.^[^ ^7^ ^]^ Furthermore, achieving high‐resolution clusters allows for pseudotemporal ordering of cells in developmental trajectory analyses,^[^ ^8^ ^]^ and provides a foundation for investigating underlying cellular interactions or communication in the context of their microenvironments.^[^ ^9^ ^]^ In immunological contexts, fine‐grained clustering supports detailed characterization of immune cell states, activation levels, and functional heterogeneity, thereby enabling deeper insights into the immune microenvironment.^[^ ^10^, ^11^ ^]^ Although scMulti‐omics methods generally perform well on datasets such as transcriptome and epigenome omics, where high dimensionality and sparsity are shared features, the distinct characteristics of RNA and antibody‐derived tag (ADT) data in CITE‐seq present unique computational challenges.^[^ ^12^, ^13^ ^]^ Specifically, RNA data exhibits high dimensionality and sparsity, whereas ADT data suffers from noise and bias.^[^ ^14^ ^]^ These contrasting data characteristics limit the effectiveness of traditional computational models on CITE‐seq data, leading to problems in precise cell clustering and functional analysis. Therefore, there is a pressing need for computational methods that can effectively extract omic‐specific information and seamlessly assemble it, enabling the discovery of valuable insights from high‐dimensional single‐cell multi‐omics datasets, especially for accurate cell clustering.

Significant advancements have been made in single‐cell clustering methods. Among them, SC3^[^ ^15^ ^]^ is a widely used integrated method that begins with gene filtering and distance matrix computation, followed by dimensionality reduction through principal component analysis (PCA) and Laplacian transformations, and ultimately applies hierarchical clustering to obtain consensus clusters. Similarly, Seurat^[^ ^16^ ^]^ offers a comprehensive suite of tools, from data preprocessing to downstream analysis, and identifies and groups cell populations by applying graph‐based algorithms. CIDR,^[^ ^17^ ^]^ on the other hand, refines the standard PCA, allowing ultrafast and accurate clustering by interpolating the scRNA‐seq data. However, these traditional approaches were designed primarily for scRNA‐seq data and rely on linear encoding strategies (e.g., PCA‐based transformations) that struggle to capture the nonlinear relationships and multimodal structure of CITE‐seq data. As a result, they fail to fully leverage complementary information provided from RNA and ADT modalities,^[^ ^18^, ^19^ ^]^ which ultimately limits their clustering performance in multimodal contexts.

Newer models have been developed specifically for CITE‐seq multi‐omics data, but each has its own technical limitations. For instance, totalVI^[^ ^20^ ^]^ employs a variational autoencoder to learn a joint probabilistic representation of RNA and ADT; while powerful, this shared latent space approach can oversmooth the data, blurring modality‐specific signals and reducing resolution in downstream interpretation. Similarly, BREM‐SC^[^ ^19^ ^]^ adopts a Bayesian random‐effects mixture model to jointly cluster RNA and ADT, but it must assume specific data distributions (e.g. Dirichlet priors) that may not hold for all datasets, hurting generality. CiteFuse^[^ ^21^ ^]^ integrates RNA and ADT by fusing their similarity matrices via similarity network fusion;^[^ ^22^ ^]^ however, this static fusion strategy assigns fixed weights to each modality, limiting adaptability when modality informativeness varies across datasets. Even Seurat's weighted nearest‐neighbor (WNN) framework^[^ ^23^ ^]^ partially addresses modality imbalance by learning relative weights for RNA and ADT, but it remains constrained by a local alignment solution – it aligns cells based on nearest‐neighbor similarity, rather than modeling more complex global cross‐modal interactions at a deeper representational level. Although these scMulti‐omics clustering methods have advanced the integration of RNA and ADT by leveraging joint embeddings or similarity matrix fusion,^[^ ^24^, ^25^, ^26^ ^]^ they still face key challenges. Many approaches integrate omics data without explicitly modeling intricate cross‐modal correlations, leading to a loss of some biologically meaningful associations.^[^ ^27^ ^]^ Moreover, methods that force a fully shared latent embedding^[^ ^28^, ^29^ ^]^ often neglect modality‐specific variation, which is crucial for capturing cellular heterogeneity.

**Figure 1:**
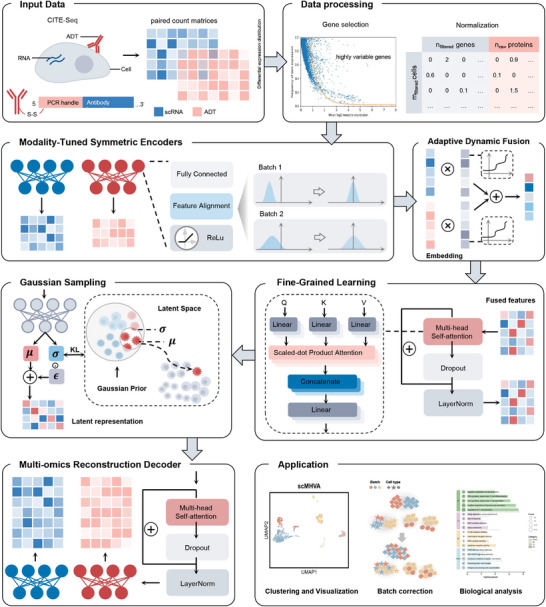
The network architecture of scMHVA. scMHVA processes paired RNA and ADT count matrices from CITE‐seq as input. The preprocessed data is passed through modality‐specific symmetric encoders to extract embeddings. An adaptive dynamic fusion module then integrates these embeddings, creating a unified view of the data. A multi‐head self‐attention mechanism captures intricate relationships within the fused features. Gaussian sampling generates latent representations that model uncertainty and create reliable data representations. Finally, a multi‐omics reconstruction decoder reconstructs the original CITE‐seq data, preserving critical information throughout the pipeline. scMHVA enables applications such as clustering and visualization, batch correction, and biological analysis.

Here, we propose scMHVA, a multi‐omics variational autoencoder model designed to cluster CITE‐seq data (**Figure** **1**). scMHVA employs two independent encoders to learn feature representations for each modality separately. An adaptive dynamic synthesis module is then applied to integrate the embeddings from both omics datasets, and this fusion is further refined using a multi‐head self‐attention mechanism to generate precise fused features. The fused features are passed through Gaussian sampling to obtain latent representation. After that, we train the model using reconstruction loss and KL divergence, with the resulting latent representation clustered using the K‐means algorithm. To evaluate the performance of scMHVA, we compared it with eight other state‐of‐the‐art single‐modal clustering algorithms and multi‐omics clustering algorithms on twelve CITE‐seq datasets. Experimental results demonstrate that scMHVA outperforms competing algorithms, delivering superior clustering accuracy and effective batch effect correction. Furthermore, scMHVA precisely delineates mouse thymocyte identities and successfully uncovers the developmental trajectories of intrathymic CD4+ T‐cell lineages.

## 2 Results

### 2.1 Overview of scMHVA Architecture

scMHVA is a deep learning framework designed to learn the common low‐dimensional latent representation of single‐cell multi‐omics (scMulti‐omics) data, particularly for CITE‐seq datasets (Figure 1). scMHVA integrates RNA and ADT data from CITE‐seq data and leverages an adaptive dynamic fusion module combined with fine‐grained learning to capture the unique characteristics and complexities of the data. The scMHVA framework has six main steps: 1) Preprocessing the raw CITE‐seq data. This includes normalizing both RNA and ADT expression matrices and selecting the highly variable genes from the RNA data. The preprocessed RNA and ADT expression matrices are then concatenated, forming the input for the subsequent steps of the model. 2) scMHVA employs a modality‐tuned symmetric encoder framework to process each modality (RNA and ADT) separately using its encoder, which can reduce the dimensionality of each omics dataset, extracting the respective embeddings while retaining essential information for downstream tasks. 3) Then, integrating the output of the RNA and ADT encoders, with an adaptive dynamic fusion module that first assigns equal weights to the data from each modality, and as the training progresses, the initial weights are dynamically adjusted for each modality, allowing the model to adaptively assign importance to RNA or ADT data depending on the context. 4) The fused features undergoes fine‐grained learning using a multi‐head self‐attention mechanism, where each attention head independently processes a subspace of the input data, allowing the model to capture diverse and complex features from specific aspects of the data. This step enhances the model's ability to discern meaningful patterns across RNA and ADT data. 5) Following this, the combined feature representation is Gaussian‐sampled through a shared encoder into a normal distribution, reducing the dimensionality further and mapping the data from different omics sources into a common latent space. 6) Finally, scMHVA learns a low‐dimensional latent representation of the scMulti‐omics data through a coarse‐to‐fine optimization training strategy. The loss function combines the reconstruction loss and the KL‐divergence loss from the variational autoencoder. The resulting low‐dimensional latent representation is suitable for tasks such as clustering and visualization, while facilitating biological interpretation of the genes and proteins, potentially uncovering dynamics of immune cell development.

### 2.2 scMHVA Exhibits Superior Clustering Accuracy and Computational Efficiency in Multi‐Omics CITE‐seq Data Analysis

We evaluated the clustering performance of scMHVA using nine real CITE‐seq datasets, which encompass both RNA data and ADT data from diverse species and organs, such as humans and mice. The size of the datasets ranges from 713 to 30 672 cells. To benchmark scMHVA, we compared it with six multi‐modal and two single‐modal clustering methods. The multi‐modal methods were scMDC,^[^ ^30^ ^]^ DeepMaps,^[^ ^31^ ^]^ BREMSC,^[^ ^19^ ^]^ MOFA+,^[^ ^32^ ^]^ totalVI,^[^ ^20^ ^]^ and Seurat.^[^ ^23^ ^]^ The single‐modal methods were scDeepCluster^[^ ^33^ ^]^ and scGAE.^[^ ^34^ ^]^ The clustering results provided by scMHVA and the compared methods on the nine real CITE‐seq datasets were evaluated by four evaluation metrics, *NMI*,^[^ ^35^ ^]^ *ARI*,^[^ ^36^ ^]^ *AMI*,^[^ ^37^ ^]^ and *ACC*.^[^ ^38^ ^]^

**Figure 2:**
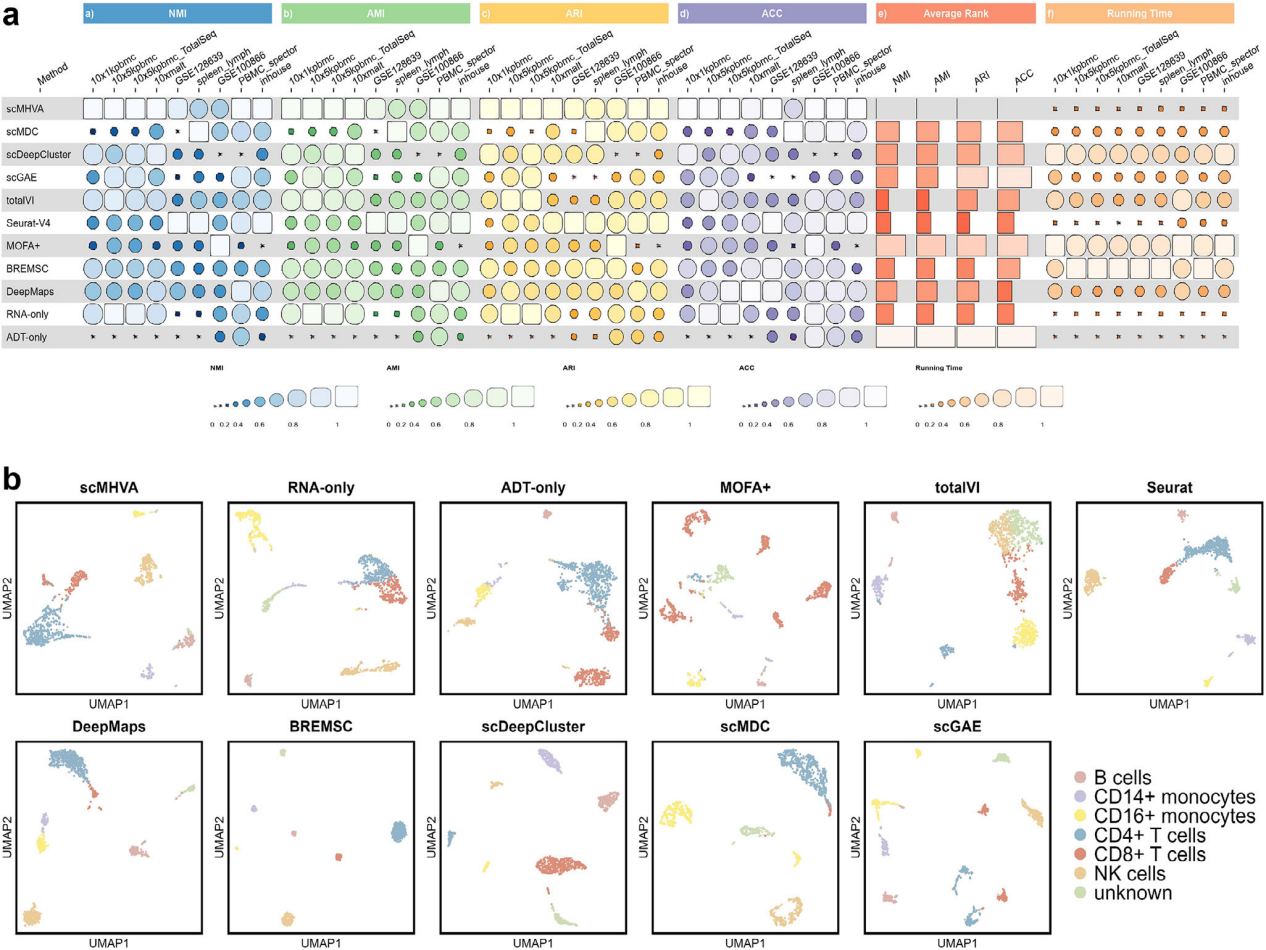
Benchmarking of scMHVA in terms of cell clustering and batch effect elimination on CITE‐seq datasets. a) Comparative evaluation of clustering performance on nine CITE‐seq datasets using NMI, AMI, ARI, ACC evaluation metrics, the average rank, and the running time for log transformations. b) UMAP visualizations of the synthesized representations of scMHVA and other competing methods on the “inhouse” dataset.

As shown in **Figure** **2**a, scMHVA consistently outperforms most multi‐modal and all single‐modal methods, achieving the highest scores on six datasets across ARI, NMI, AMI, and ACC metrics. Single‐modal methods show instability and are unable to fully exploit multi‐omics data. In contrast, scMHVA surpasses multi‐modal approaches, leveraging its fusion module to generate accurate latent representations by dynamically weighting each modality's importance. Subsequently, we ranked performance by the average of the four metric values for scMHVA and all competing clustering methods on all nine CITE‐seq datasets. As shown in Figure 2a, scMHVA consistently ranked top for all metrics, establishing its superior performance. Seurat ranked second for ARI, NMI, and AMI, and DeepMaps had the second position for ACC scores. Notably, scMHVA outperformed all deep learning‐based algorithms, highlighting the effectiveness of its multi‐head attention mechanism for analyzing CITE‐seq datasets. This advantage is particularly significant in the context of complex, multi‐modal data where the precision of feature extraction and integration is crucial. We further explored the advantages of scMHVA in handling multi‐omics data. Compared to clustering results obtained using only RNA or ADT modalities individually, scMHVA consistently achieved superior overall clustering performance when integrating both data types. This highlights its ability to effectively leverage the complementary information across modalities to produce more accurate and robust representations of cellular heterogeneity. Finally, to verify the efficiency and reproducibility of scMHVA, we conducted a comparison of scMHVA's runtime against the eight clustering methods on the nine CITE‐seq datasets. The results, illustrated in Figure 2a, indicate the faster execution times of scMHVA on all CITE‐seq datasets compared to all deep learning‐based clustering algorithms. Table S1 (Supporting Information) provides detailed statistics comparing runtime, model complexity, and architectural characteristics of scMHVA and baseline methods across multiple CITE‐seq datasets. These results indicate that scMHVA's superior efficiency among deep learning–based approaches primarily stems from its reduced parameter count and streamlined architecture, while its advantages over non–deep‐learning methods are further enhanced by leveraging GPU acceleration during training.

To evaluate the ability of scMHVA to distinguish different cell types in the latent space, we constructed UMAP plots of the predicted labels aligned with the true labels utilizing the distinct latent representation derived from scMHVA and the other clustering methods including scMDC,^[^ ^30^ ^]^ DeepMaps,^[^ ^31^ ^]^ BREMSC,^[^ ^19^ ^]^ MOFA+,^[^ ^32^ ^]^ totalVI,^[^ ^20^ ^]^ Seurat,^[^ ^23^ ^]^ scDeepCluster^[^ ^33^ ^]^ and scGAE^[^ ^34^ ^]^ on the ‘inhouse’ dataset with also the RNA and ADT data (Figure 2b) and the other eight CITE‐seq datasets (Figures S5– S8, Supporting Information). The “inhouse” dataset originated from human peripheral blood mononuclear cells, with 1372 cells divided into 7 cell types. It was processed on the 10X genomic platform using the Gel Bead Kit V2, the sequencing platform is the Illumina HiSeq, and the sequencing depth is 50 000. As seen in Figure 2b, totalVI and MOFA+ failed to effectively distinguish some of the cell types in the latent space. Specifically, totalVI barely isolated ‘unknown’ cell types and NK cells, MOFA+ appeared to generate a mixture of multiple cell types, such as CD8+ T cells, CD4+ T cells, and ‘unknown’ cell types. scMDC struggled to differentiate certain sub‐cell types, with some smaller cell clusters seemingly overshadowed by larger ones, as indicated by the lack of CD14+ monocytes, CD8+ T cells, and “unknown” cell types in the latent space. BREMSC could classify individual cell types, yet it failed to preserve the biological relationships between cells when confronted with scMHVA. scGAE generates overly fragmented clusters. Compared to the rest of the methods, scMHVA and scDeepCluster were capable of separating individual cell types in the latent space, but scMHVA better reflected the biological patterns among diverse cells within raw RNA and ADT data. For example, scMHVA distinctly differentiated NK cells, B cells, CD14+ monocytes, and CD16+ monocytes while maintaining the similarity between CD4+ T cells and CD8+ T cells. Therefore, scMHVA not only accurately segregated most cell types within the latent space but also preserved the inherent biological relationships embedded within the multi‐omics data, maintaining the fidelity of the complex interactions and patterns that define cellular heterogeneity. Taking the results together, we conclude that scMHVA enhances clustering performance through the effective latent representation derived from multi‐omics data.

### 2.3 scMHVA Excels in Multi‐Modal CITE‐seq Data Integration and Batch‐Invariant Cell Type Identification

To investigate the possible batch effect in the CITE‐seq data from the various sequencing protocols, we applied the scMHVA algorithm to process a dataset derived from bone marrow mononuclear cells (BMMCs) collected from 12 healthy donors. The dataset comprises 90 261 cells with 45 cell types and 12 batches, employing Feature Barcoding with the BioLegend TotalSeq B Universal Human Panel v1.0 for simultaneous measurement of RNA and protein expression on the Illumina NovaSeq 6000 sequencing platform.

**Figure 3:**
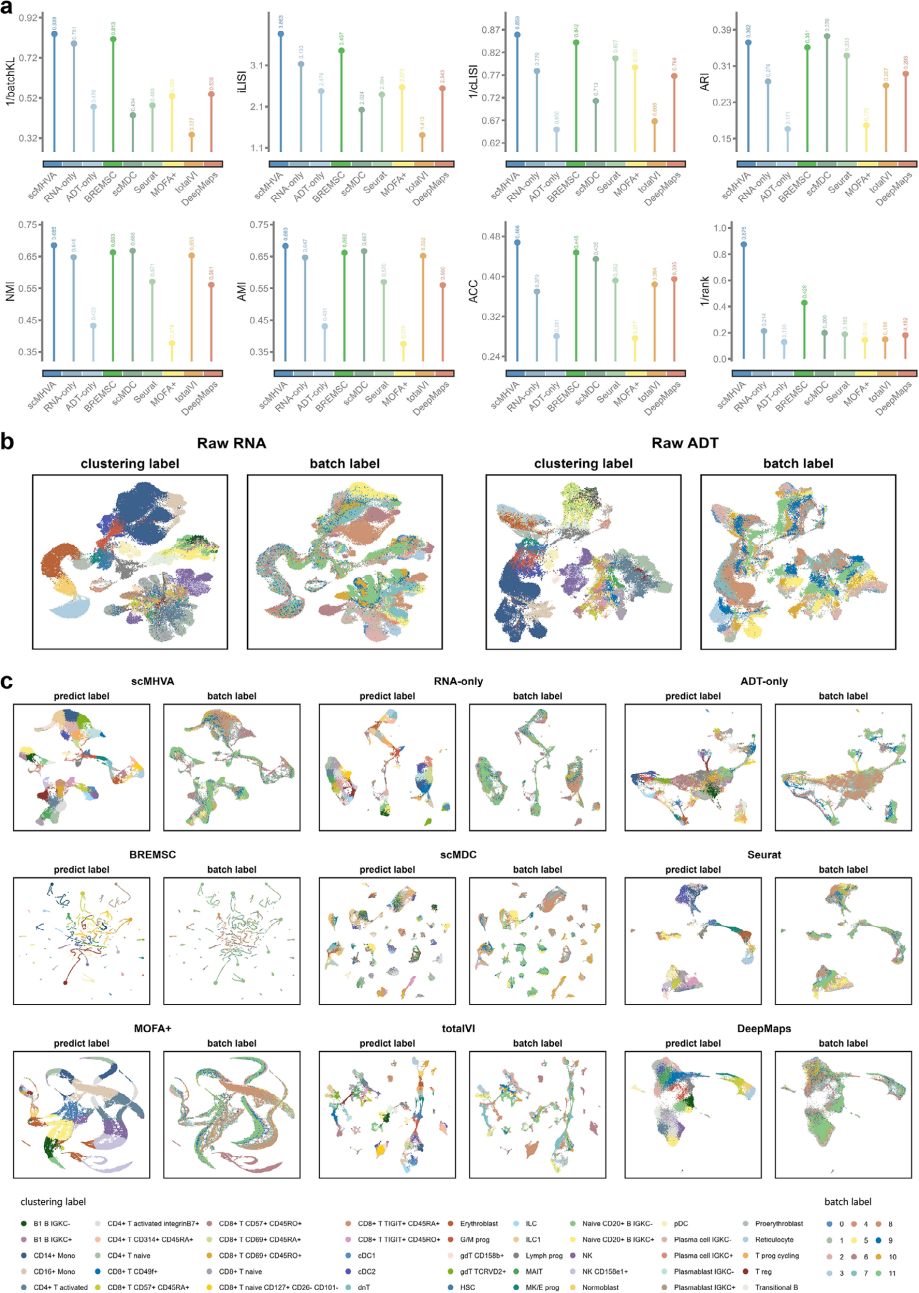
Benchmarking of scMHVA in terms of synthesized representations on multi‐omics CITE‐seq datasets. a) Analysis of batch effect elimination and clustering performance for scMHVA and the competing methods on the “GSE194122” dataset. b) UMAP visualizations of the raw RNA and ADT the ‘GSE194122’ dataset, with cells colored by clustering labels (left) and batch labels (right). c) UMAP visualizations of the latent representation of scMHVA and the competing methods on the “GSE194122” dataset, with cells colored by predict labels (left) and batch labels (right).

The clustering performance of scMHVA and other competing methods on the “GSE19412” dataset was evaluated using four evaluation metrics, as summarized in **Figure** **3** a. The results show that scMHVA consistently outperformed the baseline methods in terms of clustering accuracy. Furthermore, we employed three widely used metrics to assess batch effect correction: the Inverse Simpson's Index of Integration (iLISI), Conditional Local Inverse Simpson's Index (cLISI), and batchKL.^[^ ^39^, ^40^ ^]^ The LISI metrics evaluate integration quality (iLISI) and batch mixing (cLISI) based on local neighborhood diversity. Specifically, a cLISI value approaching one indicates excellent cell integration, an iLISI value close to the number of batches suggests effective batch mixing, and a low batchKL value signifies superior batch effect mitigation. As shown in Figure 3a, scMHVA achieved the highest iLISI and the lowest cLISI and batchKL values, demonstrating its superior ability in eliminating batch effects compared to all other baseline methods.

Figure 3b visualizes the batch effects present in the dataset. To further assess batch effect correction, Figure 3c displays the low‐dimensional latent representation generated by scMHVA and six competing methods, alongside RNA‐only and ADT‐only representations. Notably, scMHVA achieves more distinct cluster boundaries and superior batch mixing, as reflected by the uniform distribution of cells from different batches within each cluster. In contrast, several baseline methods show residual batch‐specific segregation, particularly in certain regions, indicating limited effectiveness in correcting batch effects. These comparative results highlight the strength of scMHVA in achieving accurate clustering while substantially alleviating batch‐related artifacts.

### 2.4 Hyperparameter Evaluation and Ablation Study

**Figure 4:**
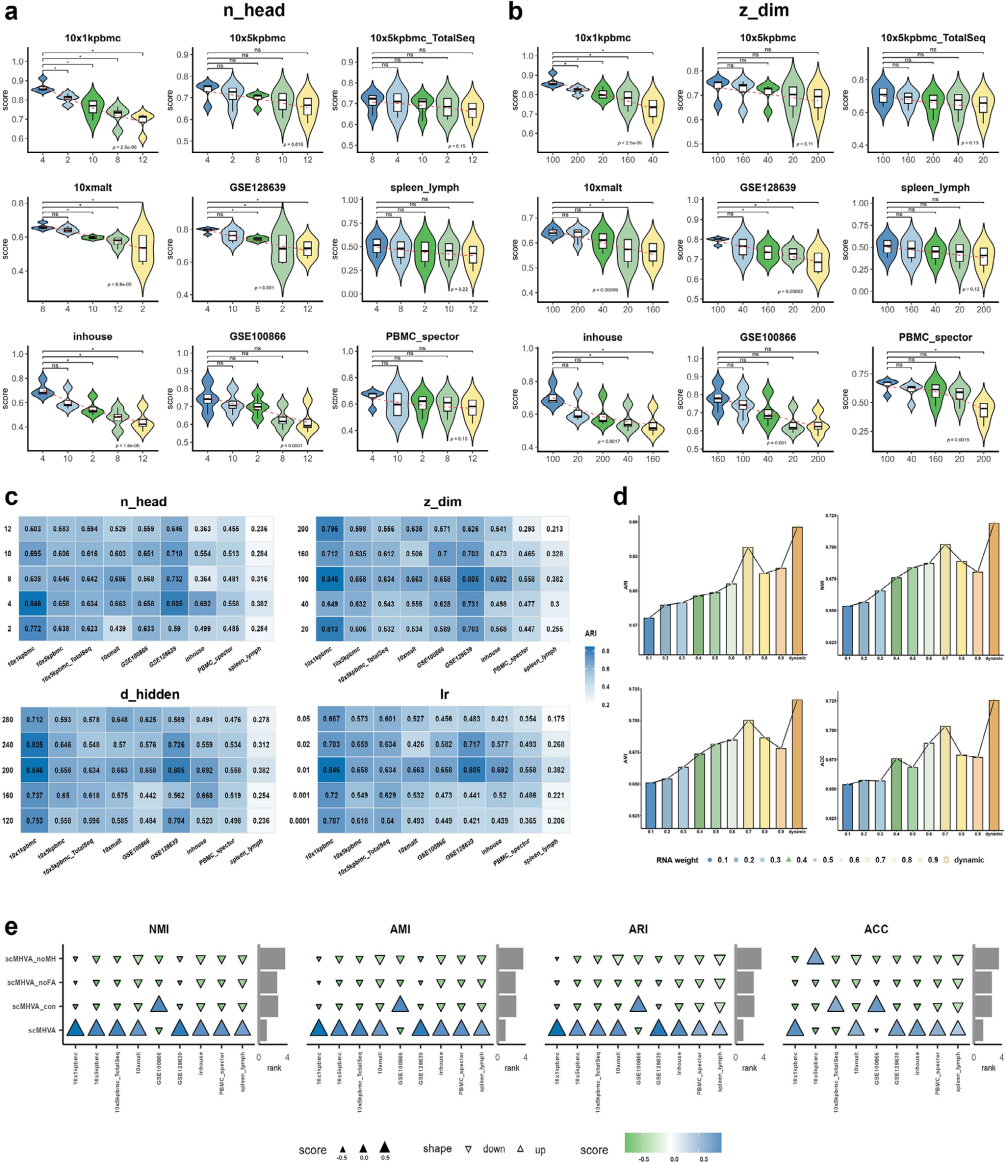
Hyperparameter tuning and ablation study of scMHVA. a,b) Effect of different numbers of attention heads (n_head) (a) and dimensions of the latent representation (z_dim) (b) on the clustering performance on CITE‐seq datasets. c) Heatmap of clustering performance across CITE‐seq datasets for different numbers of attention heads (n_head), dimensions of the latent representation (z_dim), neurons in the hidden layer (d_hidden), and learning rates (*lr*). d) Average clustering performance of nine datasets under static fusion weights. e) Clustering performance comparison between scMHVA and scMHVA without the modality fusion module and the multi‐head attention mechanism.

We performed a comprehensive analysis of the hyperparameters and components of the model to assess the effect of multiple factors on the performance of the scMHVA model. The hyperparameters included the number of attention heads (n_head), the dimension of the latent representation (z_dim), the number of neurons in the hidden layer (d_hidden), and the learning rate (*lr*). The model components evaluated were the modality fusion module and the multi‐head attention mechanism. We illustrate the clustering performance of scMHVA using different hyperparameters and model components in **Figure** **4** and Figure S9 (Supporting Information) on the nine CITE‐seq datasets measured by four evaluation metrics (NMI, ARI, AMI, ACC).

In selecting the range for each hyperparameter, we considered prior studies on multimodal and attention‐based models, preliminary empirical observations on representative CITE‐seq datasets, and practical factors such as model complexity and training stability. Specifically, the number of attention heads (n_head) was varied across {2, 4, 8, 10, 12} (Figure 4a). This parameter determines the model's ability to extract diverse feature representations in parallel. Too few heads may limit expressiveness, while too many can introduce redundancy or risk overfitting. Experimental results showed that n_head=4 achieved the best performance across most CITE‐seq datasets. For the latent dimension z_dim, we drew from common practices in deep learning models for high‐dimensional single‐cell data, testing values from {20, 40, 100, 160, 200} to balance representational capacity, clustering accuracy, and computational cost. As shown in Figure 4b, a dimension of 100 delivered the strongest clustering performance across multiple datasets. Next, we evaluated the effect of the number of neurons in the hidden layer (d_hidden), exploring values from {120, 160, 200, 240, 280}. This parameter affects the model's capacity for feature extraction. Results in Figure S9a (Supporting Information) indicate that d_hidden=200 yielded optimal performance. We also tested five settings for the learning rate (*lr*): {0.0001, 0.001, 0.01, 0.02, 0.05}, to assess their impact on training convergence and model stability. An appropriate learning rate accelerates convergence while preventing divergence or oscillation. As shown in Figure S9b (Supporting Information), *lr* = 0.01 consistently produced the best clustering results. Finally, quantitative comparison of clustering performance (ARI, NMI, ACC, and AMI) using different numbers of highly variable genes (HVGs): 300, 500, 1000, 1500, and 2000. Results across nine CITE‐seq datasets show that performance remains relatively stable across thresholds, with the best overall accuracy achieved when selecting the top 500 HVGs (Figure S9c, Supporting Information). Based on these considerations, we systematically explored the hyperparameter space using a grid search strategy and performed sensitivity analyses to confirm the stability and robustness of the chosen settings (Figure 4c; Figures S10– S12, Supporting Information).

In addition, we conducted a comprehensive evaluation of static fusion strategies across nine CITE‐seq datasets to highlight the necessity of our adaptive fusion mechanism. Specifically, we varied the weights assigned to the RNA and ADT modalities from 0.1 to 0.9. Figure 4d shows the average clustering performance across the nine datasets under different fusion weight configurations, and the detailed clustering results for each dataset are provided in Table S3 (Supporting Information). In static fusion, fixed weights are uniformly assigned to each modality across all samples and datasets. However, different fixed‐weight settings lead to significant variations in clustering performance, and no single fixed configuration achieves consistently optimal performance across diverse biological contexts or tissue types, underscoring the inherent limitations of static fusion. In contrast, our adaptive dynamic fusion mechanism adjusts weights in a data‐driven manner and consistently outperforms all static fusion configurations. Moreover, the RNA and ADT weights learned by our model vary across datasets (Figure S13b, Supporting Information), demonstrating its ability to automatically learn optimal fusion weights based on the input data.

To investigate whether the key components of scMHVA contribute to improved clustering performance on CITE‐seq data, we conducted a series of ablation studies. Specifically, we compared scMHVA to three ablated variants: scMHVA_con, scMHVA_noMH, and scMHVA_noFA. Here, scMHVA_con denotes our model using concatenated RNA and ADT inputs as a single modality, scMHVA_noMH refers to the model without the multi‐head attention mechanism, and scMHVA_noFA is the model without the feature alignment layer. In Figure 4e, the triangle area reflects the magnitude of each clustering metric, while upward and downward triangles indicate whether a method performs better or worse than other methods on the corresponding dataset; blue represents higher scores, and green represents lower scores. We can observe that investigating the model without an attention mechanism, scMHVA surpassed scMHVA_noMH in clustering across all of the CITE‐seq datasets, demonstrating the essential role of the multi‐head attention mechanism for enhancing the clustering performance of scMHVA. For the case investigating using the concatenated RNA and ADT data, Figure 4e shows that scMHVA exhibited consistently superior performance over scMHVA_con across almost all CITE‐seq datasets. Except for the ‘GSE100866’ dataset, where scMHVA_con obtained clustering results similar to those of scMHVA. However, the remaining datasets showed that integration of the concatenated fusion module led to a decline in the clustering performance of scMHVA. The performance improvement between scMHVA and scMHVA_con was significant, indicating that the modality fusion module is an effective fusion module to integrate diverse modalities from multi‐omics data, especially from CITE‐seq datasets. Futhermore, removing the feature alignment layer led to a consistent decline in performance across all evaluation metrics and datasets. This highlights the critical role of the feature alignment layer in integrating modality‐specific features into a common latent space, thereby improving the model's capability to integrate multi‐modal inputs.

### 2.5 scMHVA Enables Accurate Clustering on Large‐Scale CITE‐seq Datasets

**Figure 5:**
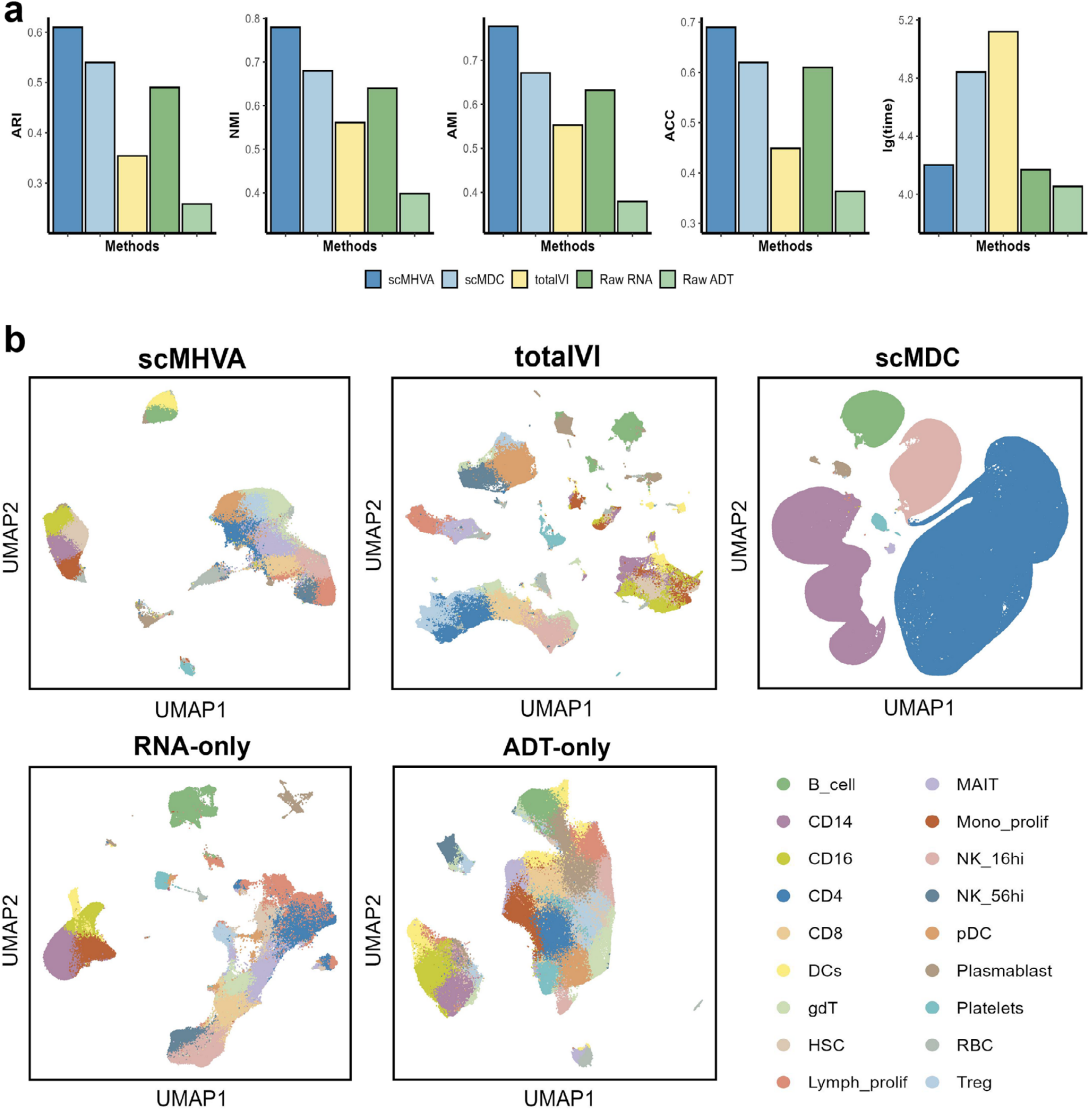
Scalability evaluation of scMHVA based on the Haniffa COVID Dataset. a) Comparative evaluation of clustering performance on the Haniffa COVID dataset using ARI, NMI, AMI, and ACC evaluation metrics and the running time for log transformations. b) UMAP visualizations of the latent representation of scMHVA and the competing methods on the Haniffa COVID dataset.

We applied the scMHVA algorithm to analyze the Haniffa COVID Dataset,^[^ ^41^ ^]^ aiming to evaluate its scalability and effectiveness for clustering analysis in large‐scale multi‐omics datasets. The dataset comprises 18 cell types and 647,366 cells, with simultaneous measurements of transcriptomes and surface proteins. As illustrated in **Figure** **5**a, we conducted a comparative evaluation of clustering results obtained using scMHVA, totalVI, scMDC, and raw RNA and ADT data. Notably, six additional state‐of‐the‐art methods failed to process this dataset due to scalability limitations, and their results were therefore omitted from the comparison. Among the evaluated methods, scMHVA exhibited consistently superior clustering performance, achieving the highest scores across multiple standard metrics, including ARI, NMI, AMI, and ACC. Importantly, scMHVA maintained a relatively short runtime compared to the other methods, and compared to directly processing RNA and ADT data separately, the runtime of scMHVA did not increase significantly.

To further assess the quality of the learned latent representation, we visualized the latent representation learned by each method using UMAP in two dimensions. As shown in Figure 5b, it is evident that the latent representation visualization using the predicted labels generated by scMHVA distinguishes cell types more clearly than other competing methods. Specifically, totalVI, RNA‐only, and ADT‐only approaches exhibited mixed latent representation with overlapping cell types, while scMDC struggled to distinguish sub‐cell types, with smaller clusters being overshadowed. This indicates their limited ability to discriminate cellular subpopulations. In contrast, scMHVA produced clearly defined and well‐separated clusters corresponding to distinct cell types, demonstrating its ability to preserve fine‐grained biological differences and effectively integrate heterogeneous omics data. These findings highlight the robustness and scalability of scMHVA in processing complex multi‐omics datasets and delivering superior clustering results, making it a powerful tool for single‐cell multi‐modal analysis.

### 2.6 scMHVA Accurately Identifies Cell Types and Reveals Dynamics of Immune Cell Development Through Multi‐Modal CITE‐seq Analysis

We present a case study in which scMHVA is applied to a published mouse thymocyte dataset^[^ ^42^ ^]^ to demonstrate its capability in modeling CITE‐seq data for accurate characterization of cell identities and successfully uncovering the developmental trajectories of intrathymic CD4+ T cell lineages.

**Figure 6:**
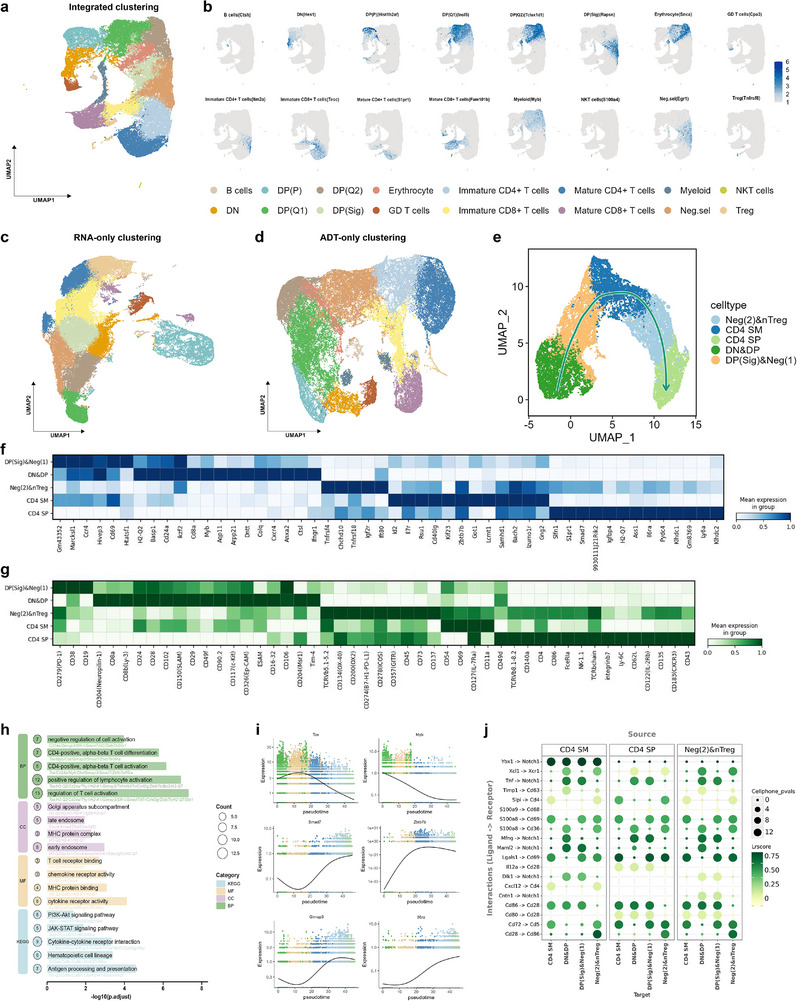
scMHVA reveals cellular interactions and immune signaling pathways in multi‐omics CITE‐seq data. a) UMAP for scMHVA cell clustering results from integrated RNA and ADT data. Cell clusters were annotated based on marker genes and ADTs. b) Feature plot of marker RNA expression levels for each cell cluster identified by scMHVA integrated clustering. c‐d UMAPs for scMHVA cell clustering results from RNA‐only (c). and ADT‐only (d). e) Pseudotime analysis visualization of the CD4+ T cell lineage. f,g) Heatmap of marker RNA (f) and marker ADT (g) expression levels for each cell cluster in the CD4+ T cell lineage. h) Gene enrichment analysis of the CD4+ T cell lineage. i) Visualisation of expression levels of genes related to the “CD4‐positive, alpha‐beta T cell differentiation“ pathway. j) Cell‐cell communication analysis of the CD4+ T cell lineage.

First, we applied scMHVA to integrate CITE‐seq data (72,042 cells) and performed clustering and visualization based on RNA and ADT information. scMHVA successfully identified 16 distinct cell types. Based on the identified cell clusters, we detected and visualized the expression levels of marker genes and marker ADTs for each cluster ( **Figure** **6** b; Figures S16– S21, Supporting Information). We annotated each cluster using these known marker genes and ADTs, resulting in 16 distinct thymocytes, including CD4^‐^CD8^‐^ (DN), proliferating DP, quiescent pre‐selection DP, post‐TCR‐recombination DP receiving positive selection signals (DP (Sig)), immature and mature CD4+ and CD8+ T cells, negative selection populations, along with B cells, erythrocyte, myeloid, regulatory (Treg), natural killer T cells (NKT), and GD T cells (Figure 6a). Compared to cell types identified using only RNA (Figure 6c) or ADTs (Figure 6d), scMHVA distinctly separates and annotates cell populations that neither modality could resolve on its own. For instance, RNA‐only clustering failed to distinguish immature CD4+ T cells, while ADT‐only clustering could not resolve DP (Sig) cells. By integrating both RNA and ADT modalities, scMHVA accurately identifies these biologically meaningful subsets, demonstrating its superior ability to dissect complex cellular heterogeneity within CITE‐seq datasets. We also compared the clustering performance of scMHVA with other multi‐omics methods on this CITE‐seq data. As shown in Figure S22 (Supporting Information), scMHVA consistently achieved the highest performance.

To further explore scMHVA's ability to integrate dual‐modality information from CITE‐seq datasets, we examined its capacity to characterize cellular heterogeneity at the finer scale of cell subtypes. We isolated CD4+ T cells spanning from the double‐negative (DN) to single‐positive (SP) stages, performed separate clustering, and then applied Monocle3^[^ ^43^ ^]^ for pseudotime analysis and visualization (Figure 6e). Specifically, we used the ‘FindAllMarkers’ function from the “Seurat” package^[^ ^23^ ^]^ to identify marker genes and marker ADTs for the individual cell clusters, as shown in Figures 6f and 6g. Based on these marker genes and ADTs, we annotated the cells and identified five developmental stages: the double‐negative and early double‐positive populations (DN&DP), the population undergoing positive selection and partially early negative selection (DP(Sig)&Neg(1)), semimature CD4+ T cells (CD4 SM), the population undergoing late negative selection with partial differentiation into nTregs (Neg(2)&nTreg), and mature CD4+ T cells (CD4 SP). The dynamic changes in gene expression levels along the inferred pseudotime are shown in Figure S23 (Supporting Information), including early downregulation of Rag1, sustained downregulation of early markers Ccr9 and Cd24a, and late upregulation of maturation markers such as Klf2 and Slfn1, among others.^[^ ^44^ ^]^

Moreover, to better understand the functional significance of the embedded representations synthesized by scMHVA in the development of CD4+ T cell lineage, we performed gene enrichment analysis using the Gene Ontology (GO)^[^ ^45^ ^]^ and Kyoto Encyclopedia of Genes and Genomes (KEGG)^[^ ^46^ ^]^ databases. With the “clusterProfiler” package,^[^ ^47^ ^]^ we selected the top 20 most highly expressed genes for each cell type identified by scMHVA. These genes were then subjected to GO and KEGG enrichment analysis to uncover their functional enrichment in biological processes, revealing potential signaling pathways and cellular functions. Figure 6h displays the enrichment level of genes in our analysis. We identified pathways closely associated with intrathymic CD4+ T cell differentiation, such as ‘CD4‐positive, alpha‐beta T cell differentiation’. The expression dynamics of the key genes involved in this pathway over pseudotime are shown in Figure 6i, among which Tox is an indispensable transcription factor in CD4+ T cell lineage commitment. It is upregulated following TCR signaling activation in the thymus, particularly during the positive selection phase of double‐positive (DP) thymocytes,^[^ ^48^ ^]^ and it promotes CD4+ T cell differentiation by regulating the expression of THPOK (encoded by Zbtb7b in mice).^[^ ^49^ ^]^

Finally, we performed cell‐cell communication analysis to further explore this biological process. We used LIANA, a ligand‐receptor analysis framework as an open‐source interface to all the resources and methods,^[^ ^50^ ^]^ which provides methods for inferring cell‐cell communication from CITE‐seq data. Figure 6j shows the ligand–receptor interactions between RNA and protein modalities. We found that the interaction ‘Cd86‐Cd28’ had a high communication score. Development and central tolerance of T lymphocytes in the thymus requires both TCR signals and collaboration with signals generated through co‐stimulatory molecule interactions.^[^ ^51^ ^]^ As a classic co‐stimulatory signaling pathway, “Cd86‐Cd28” interaction provides the second signal required for T cell differentiation and plays a role in both thymic CD4+ T cell selection and peripheral immune function.^[^ ^52^, ^53^ ^]^ Furthermore, previous studies have shown that Cd28 co‐stimulatory signaling is indispensable for the differentiation and maintenance of regulatory CD4+ T cells (nTregs).^[^ ^54^, ^55^, ^56^ ^]^ In our analysis, this interaction primarily acted at the late stage of negative selection (Neg(2)), where CD4+ T cells with high‐affinity peptide recognition received Cd28 co‐stimulation to initiate nTreg development, thereby escaping negative selection and suppressing apoptosis. In summary, analysis of thymic T‐cell development demonstrates that scMHVA effectively integrates bioinformatic signals from CITE‐seq data to characterize cellular heterogeneity and uncover meaningful biological insights.

## 3 Discussion

In this study, we propose scMHVA, a lightweight model that leverages a multi‐head attention mechanism for the analysis of CITE‐seq data. scMHVA applies modality‐tuned symmetric encoders to obtain embeddings and uses z‐score normalization to smooth the influence of noise in the features. It effectively integrates RNA and ADT embeddings through an adaptive dynamic fusion module, which dynamically assigns adaptive weights to each modality of the CITE‐seq data. Subsequently, scMHVA employs a multi‐head attention mechanism to capture information from the fused features, a critical step in enhancing model performance and improving generalization. To learn the latent representation of multi‐omics CITE‐seq data, scMHVA optimizes both the combined reconstruction loss and the KL‐divergence loss simultaneously.

To validate the effectiveness of scMHVA in clustering the CITE‐seq data, we conducted a comparison of scMHVA with six scMulti‐omics clustering methods and two scRNA‐seq clustering methods. The experimental results highlight the effectiveness of our model in integrating and characterizing different paired RNA and ADT data originating from various sources. Specifically, scMHVA demonstrated an outstanding capability to identify subgroups that are not usually distinguishable through alternative approaches, while maintaining the inherent relationships within the subgroups. Furthermore, scMHVA demonstrated proficient elimination of batch effect and superior computation efficiency across different CITE‐seq datasets compared to other clustering algorithms. The clustering results are crucial for the downstream analyses of scMulti‐omics data. In the integrated analysis of single‐cell gene expression and ADT data, scMHVA precisely identified and annotated sixteen distinct cell types within a CITE‐seq dataset from mouse thymocyte cells using marker genes and ADTs. Our model enhanced the resolution of cell type identification and provided a comprehensive view of cellular heterogeneity by clustering RNA and ADT jointly. Furthermore, we investigated scMHVA's capacity to characterize cellular heterogeneity at the finer scale of cell subtypes. Through the analysis of thymic T cell development, we demonstrated the functional significance of the latent representation learned by scMHVA, which provided deeper insights into cellular heterogeneity and enabled the discovery of meaningful biological interpretations.

Taking all these findings together, we have demonstrated that scMHVA effectively learns synthesized representations from single‐cell CITE‐seq data, enabling comprehensive characterization of cellular heterogeneity and interpretation of underlying regulatory mechanisms. Through its innovative deep learning‐based architecture, scMHVA not only excels in technical aspects such as batch effect removal and clustering accuracy but also provides meaningful biological insights into cell type‐specific features and molecular interactions. In the future, we will extend scMHVA's capabilities to accommodate emerging multi‐omics modalities and handle atlas‐scale datasets to further advance our understanding of complex cellular systems through integrated data analysis.

## 4 Experimental Section

### Preliminaries

The development of CITE‐seq technology has enabled the simultaneous profiling of transcriptomic and proteomic information at the single‐cell level. Such multi‐modal data provides new insights into cellular heterogeneity and function. A central challenge in analyzing this type of data lies in effectively integrating these diverse sources of information. To address this, a model is designed that extracts, fuses, and aligns information from multiple modalities, projecting it into a unified representation space to support downstream tasks such as clustering.

To ensure terminological clarity and consistency throughout this work, the following key concepts used in the model framework is defined:

- **Embedding**: This term refers to the intermediate feature representation extracted from each input modality using modality‐specific encoders. These embeddings capture the essential information of the raw input data within their respective modalities in a lower‐dimensional space and serve as the basis for subsequent fusion.
- **Latent representation**: This term denotes the final variable sampled from the latent space. It is obtained by sampling from a Gaussian distribution parameterized by a mean and variance derived after the fusion of embeddings. The latent representation serves as the compact, integrated code used for downstream tasks such as clustering and visualization.
- **Fused features**: This term describes the output of the modality fusion module, which integrates the embeddings from different modalities into a unified feature representation.

### Processing and Normalizing Data

The raw CITE‐seq data **X**, comprising RNA (**X**_*R*_) and ADT (**X**_*A*_) data, is preprocessed using the Scanpy package.^[^ ^57^ ^]^ Key steps include: 1) selecting the top *d* (500) highly ranked genes from RNA data and all ADT features to retain informative features; 2) removing zero‐count genes to mitigate high dropout rates; 3) normalizing cell counts to correct for technical variability; and 4) applying a logarithmic transformation and scaling to unit variance and zero mean. The processed RNA and ADT data are denoted as $X_{r}$ and $X_{a}$ in the model.

### Modality‐Tuned Symmetric Encoders

In CITE‐seq, both transcriptomic and ADT modalities originate from the same pool of cells, but differ substantially in feature dimensionality: transcriptomic profiles typically encompass tens of thousands of genes per cell, whereas ADT panels measure only a few hundred protein markers. Moreover, unlike scRNA data, which are inherently sparse, ADT data are dense, further highlighting the distinct characteristics of these two modalities. To address these challenges, modality‐tuned symmetric encoders were employed.

Specifically, the preprocessed RNA data $X_{r}$ and ADT data $X_{a}$ as inputs were first adopted to the modality‐specific encoder to extract their respective hidden representations *h*_*r*_ and *h*_*a*_:

(1) $$h_{r/a}=f\left(g\left(W_{r/a}X_{r/a}\right)+b_{r/a}\right)$$

where $X_{r/a}$ is the RNA or ADT modality for the CITE‐seq data, *W*_*r*/*a*_ is the weight matrix of the respective encoder layers of the RNA or ADT modality, which is initialized as a learnable parameter. *b*_*r*/*a*_ is the bias vector of the encoder. The function f(·) is a non‐linear activation function, here the RELU function was used. The g denotes the feature alignment layer, represented as:

(2) $$h_{r/a}=z\_score\left(h_{r/a}\right)*\gamma +\beta .$$

The process comprises two main steps. First, feature values undergo z‐score normalization, defined as $\frac{h-E[h]}{\sqrt{Var[h]}+\varepsilon }$, where *E*[*h*] and *Var*[*h*] denote the mean and variance of *h*, respectively, and ϵ represents a small constant added for numerical stability. This normalization locally smooths the influence of noise by centering the features around zero and scaling them to have a unit variance. Consequently, the amplification effect of noise within the absolute value range is mitigated, thereby reducing the impact of variable sequencing depths across cells.^[^ ^58^, ^59^ ^]^ Furthermore, z‐score normalization highlights dominant expression patterns while suppressing irrelevant variations frequently observed in CITE‐seq data.^[^ ^60^ ^]^ Following normalization, linear scaling and translation are applied to the features, with γ and β representing the scaling and translation parameters, initialized to 1 and 0, respectively. This approach simplifies information integration across both modalities by aligning RNA and protein features onto a common scale, enabling the model to capture cellular heterogeneity and relationships more effectively.

### Adaptive Dynamic Fusion of Heterogeneous Modality Information

After extracting the features of mRNA and ADT and mapping them to the same latent space, an adaptive dynamic fusion module designed to effectively integrate information from different modalities was introduced. Single‐omics data captures cell identity from a singular perspective, whereas the integration process aggregates cell‐specific information from diverse omics layers to construct a comprehensive representation of the cell. This dynamic weighting scheme enables the model to adaptively prioritize the heterogeneous information of each modality, thereby providing a more accurate reflection of their respective importance in the clustering process. By learning these weights, the model not only improves clustering precision but also fosters a deeper understanding of the underlying multi‐omics data structure. This module facilitates a data‐driven optimization of the clustering algorithm's sensitivity to varying omic datasets, ensuring the model's focus is aligned with the most informative signals within the multi‐omics data. It can be defined as follows:

(3) $$\begin{array}{cc} y=\; & w_{r}h_{r}+w_{a}h_{a}\\ w_{r/a}=\; & \frac{exp{\left(w\right)}_{r/a}}{exp\left(w_{r}\right)+exp\left(w_{a}\right)} \end{array}$$

where *y* is the integrated latent representation, *w*_*r*/*a*_ is the weight matrix of the RNA or ADT modality for the CITE‐seq data, each input modality is given the same weight of 0.5 initially, and then the weight *w*_*r*/*a*_ is updated by softmax function for normalization, *exp*(·) function represents the value of the exponential function to ensure that the real input data can be converted to a positive output.

### Fine‐Grained Learning for Cellular Embedding Representation

The fused features, while integrating information from multiple modalities, may lack the ability to capture intricate inter‐modal dependencies. To address this limitation, a multi‐head attention mechanism was incorporated, which enhances the model's capacity to effectively capture the intrinsic structures and unique characteristics of the CITE‐seq data. Specifically, this module enables interactive learning between features by computing attention scores between elements at distinct locations. The fused features $y\in R^{B\times D}$ along the feature dimension into *L* embedding subspaces, resulting in $\hat{y}\in R^{B\times L\times d_{l}}$, where *B* denotes the batch size, *D* is the dimensionality of the fused features, *L* denotes the number of partitioned feature subspaces, and *d*_*l*_ = *D*/*L* represents the feature dimensionality of each subspace. This representation enables the multi‐head attention mechanism to perform fine‐grained modeling of different feature subspaces within a single cell, thereby capturing more comprehensive intrinsic structural information. Queries, keys, and values are generated through linear transformations and are split into multiple attention heads:

(4) $$q=Split\left(\hat{y}W_{q}\right),k=Split\left(\hat{y}W_{k}\right),v=Split\left(\hat{y}W_{v}\right)$$

where $W_{q},W_{k},W_{v}\in R^{d_{l}\times d_{l}}$ are projection matrices, the Split operation divides the feature dimension of the linearly transformed matrix $\hat{y}W_{q},\hat{y}W_{k},\hat{y}W_{v}\in R^{B\times L\times d_{l}}$ evenly into *n* attention heads, each with dimensionality *d* = *d*_*l*_/*n*. In each attention head, a self‐attention scoring mechanism is employed, where weights are assigned based on the similarity scores computed between query and key pairs. These scores are normalized using the softmax function, ensuring a probabilistic interpretation. A weighted sum of the values is then calculated, enabling the model to prioritize the most relevant features and improve the quality of the learned representations. Subsequently, the outputs from all *n* heads are concatenated, passed through a linear transformation, and flattened to produce the output $y^{\prime }\in R^{B\times D}$. To further stabilize the model and improve its performance, residual connections are applied to $\hat{y}$, followed by Layer Normalization, ensuring a robust and consistent framework:

(5) $$\begin{array}{c} y_{i}^{\prime }=softmax\frac{q_{i}k_{i}^{T}}{\sqrt{d}}v_{i}=\left(\frac{e^{q_{i}k_{i}^{T}/\sqrt{d}}}{\sum_{l=1}^{L}e^{q_{i}k_{i}^{T}/\sqrt{d}}}\right)v_{i} \end{array}$$

(6) $$y^{\prime }=[y_{1}^{\prime }\;∥\;y_{2}^{\prime }\;∥\;\cdots \;∥\;y_{n}^{\prime }]w_{o}$$

(7) $$y^{\prime }=N\left(\hat{y}+D\left(y^{\prime }\right)\right)$$

where $q_{i},k_{i},v_{i}\in R^{B\times L\times d}$ are the query, key, and value matrices of the *i*th attention head (*i* ∈ {1, 2, …, *n*}, *n* is the number of heads), respectively, *T* is the transpose operation on a matrix and *d* is the feature dimensionality of each attention head used to scale the dot‐product attention scores to prevent the gradient from vanishing or exploding, $y_{i}^{\prime }\in R^{B\times L\times d)}$ is the output of *i*th attention head, [· ‖ ·] denotes concatenation along the feature dimension, and $w_{o}\in R^{d_{l}\times d_{l}}$ is the output weight matrix, it maps the integrated vectors to the desired output dimensions as the input to the encoder. N(·) denotes the layer normalization operation, and D(·) denotes the dropout operation. Following the refinement of the fused features, Gaussian sampling was further introduced to enhance the robustness and diversity of the latent representation. CITE‐seq data from different modalities may exhibit significant distributional differences. By applying Gaussian sampling, a unified perturbation is introduced to the representations of these modalities within the latent space, preventing the space from becoming overly discrete or constrained. This enables the model to better capture complex patterns and inter‐modality relationships, thus improving the separability and expressive power of the latent representation. Specifically, $y^{\prime }$ is encoded into a Gaussian‐distributed $z\in R^{B\times Z}$, Where *Z* is the feature dimension of the latent representation, as follows:

(8) $$z∼N(\mu ,\sigma^{2}I),$$

where μ represents the mean, σ represents the standard deviation controlling the degree of diversity, and I represents the identity matrix. However, directly sampling from Equation (8) would prevent the error from being backpropagated through the network for parameter updates. To overcome this, the reparametrization trick was utilized, as expressed by:

(9) z=μ+σε,

where $\varepsilon \in N\left(0,I\right)$. This approach allows both the mean and standard deviation to be learned by the network. Specifically, the mean and variance are calculated as:

(10) $$\mu =D(g\left(W_{\mu }y^{\prime }\right)+b_{\mu }),$$

(11) $$log\left(\sigma^{2}\right)=D\left(g\left(W_{\sigma }y^{\prime }\right)+b_{\sigma }\right),$$

where D(·) denotes the dropout operation, g(·) is a feature alignment layer, and $W_{\mu },W_{\sigma }\in R^{D\times Z}$, $b_{\mu },b_{\sigma }\in R^{Z}$ are the weight matrices and bias vectors for calculating the mean and variance, respectively.

### Multi‐Omics Reconstruction Decoder

To enhance the expressive ability of the latent representation z, in the decoder, the same multi‐head self‐attention mechanism is applied to the latent representation z to improve the process of reconstructing multi‐omics data, and then a modality‐specific decoder was used to map the z into the original multi‐omics data space. The structure of the decoder is symmetric to that of its corresponding encoder, intending to reconstruct the original CITE‐seq data, including RNA data and ADT data. The multi‐omics reconstruction decoder can be denoted as follows:

(12) $$z^{\prime }=Multi\_Attn\left(zW_{q}^{\prime },zW_{k}^{\prime },zW_{v}^{\prime }\right)$$

(13) $$z^{\prime }=N\left(z+D\left(z^{\prime }\right)\right)$$

(14) $${\hat{X}}_{r/a}=f^{\prime }\left(g^{\prime }\left(W_{r/a}^{\prime }z^{\prime }\right)+b_{r/a}^{\prime }\right)$$

where Multi_Attn refers to the process of obtaining a more expressive latent representation $z^{\prime }$ through the multi‐head self‐attention mechanism, $W_{q}^{\prime }$, $W_{k}^{\prime }$, and $W_{v}^{\prime }$ are projection matrices. N(·) denotes the layer normalization operation, and D(·) denotes the dropout operation. ${\hat{X}}_{r/a}$ denotes the reconstructed RNA data or ADT data, the *f*′(·) function denotes the fully connected layer, which is responsible for reconstructing the features corresponding to the omics data, and the *g*′(·) function denotes feature alignment layer. $W_{r/a}^{\prime }$ and $b_{r/a}^{\prime }$ are the weights and biases of the fully connected layer of each modality. These parameters are then updated by computing the gradient of the loss function during backpropagation.

### Coarse‐To‐Fine Optimization Training

The training of the clustering model is divided into two phases: coarse pretraining and fine training.

During the pretraining phase, the autoencoder was trained to learn low‐dimensional representations from high‐dimensional CITE‐seq data. The goal of pretraining is to minimize the difference between the reconstructed data and the original data, namely, the reconstruction loss:

(15) $$L_{pre}=L_{rec}=L_{rec}^{RNA}+L_{rec}^{ADT}$$

where $L_{rec}^{RNA}$ and $L_{rec}^{ADT}$ denote the reconstruction loss for the different modalities of CITE‐seq data, and they are calculated as follows:

(16) $$L_{rec}=\frac{1}{2}\left({\left(X_{r}-{\hat{X}}_{r}\right)}^{2}+{\left(X_{a}-{\hat{X}}_{a}\right)}^{2}\right)$$

During the training phase, pre‐trained weights was utilized to better learn the latent representation of the CITE‐seq data. The reconstruction loss (16) and KL divergence loss (18) are incorporated to impose constraints on the distribution of latent variables:

(17) $$L_{train}=L_{rec}^{RNA}+L_{rec}^{ADT}+\alpha L_{kl}$$

(18) $$L_{kl}=KL\left[N\left(\mu ,\sigma^{2}\right),N\left(0,1\right)\right]$$

where α is a hyperparameter used to balance the contribution between the KL divergence loss and the reconstruction loss, α was set to 0.0001.

### Model Implementation

A deep learning framework was introduced, scMHVA, tailored for the analysis of CITE‐seq data, leveraging the unique characteristics of such datasets for further insights. The number of hidden layer neurons of the encoder was set to 200 for both RNA and ADT data, the dimension of the latent space was set to 100, and the number of self‐attentive heads was set to 4. The Adam optimizer with a learning rate of 0.01 was used during the pretraining and training processes. Fifty epochs were pretrained to optimize the reconstruction loss and trained 100 epochs to jointly optimize the reconstruction loss and KL divergence loss. The K‐means clustering method was performed on the learned latent representations using the true number of clusters *K* for each dataset.

### Competing Methods

- scMDC^[^ ^30^ ^]^ (https://github.com/xianglin226/scMDC). It is an integrated deep multimodal autoencoder, which uses an encoder to fuse diverse modal data and twin decoders to restore each modality individually. scMDC clearly distinguishes the origin of the data and jointly learns hidden embeddings for clustering insights.
- DeepMaps^[^ ^31^ ^]^ (https://github.com/OSU‐BMBL/deepmaps). It is a tool for inferring biological networks from scMulti‐omics data. DeepMaps represents scMulti‐omics as a heterogeneous graph and employs a multi‐head graph transformer to learn cell‐gene interactions in localized and broad contexts robustly.
- BREMSC^[^ ^19^ ^]^ (https://github.com/tarot0410/BREMSC). It is a Bayesian Random Effects Mixture model integrating MCMC and Dirichlet distributions, designed to cluster paired single‐cell CITE‐seq data simultaneously.
- totalVI^[^ ^20^ ^]^ (https://github.com/YosefLab/totalVI_reproducibility). It is a deep generative tool for CITE‐seq data, learning a joint probability representation that considers unique noise, biases, and batch effects from each data modality.
- MOFA+^[^ ^32^ ^]^ (https://github.com/bioFAM/MOFA2). It is a Bayesian group factor analysis framework to integrate multi‐omics data, inferring a compact representation through a few latent factors that capture key variability sources.
- Seurat(V4)^[^ ^23^ ^]^ (https://github.com/satijalab/seurat). It is an R toolset for analyzing multi‐omics data, using an unsupervised approach with weighted nearest neighbors to determine modality “weights” unique to each cell. This enhances the integration of various types of data in analysis.
- scDeepCluster^[^ ^33^ ^]^ (https://github.com/ttgump/scDeepCluster). It is a deep zero‐inflated negative binomial (ZINB)‐based clustering method designed for scRNA‐seq data, which efficiently maps read count matrices into low‐dimensional representations.
- scGAE^[^ ^34^ ^]^ (https://github.com/ZixiangLuo1161/scGAE). It is a dimensionality reduction technique that maintains the topological integrity of scRNA‐seq data by constructing a cellular graph and employing a multitask graph autoencoder to retain topological and feature details concurrently.

### Benchmarking Setup

To ensure fair comparison and reproducibility, hyperparameter tuning was conducted for all baseline methods used in the experiments. For each baseline model, key hyperparameters were first identified that are known to influence its performance. The search ranges for these hyperparameters were selected by moderately adjusting the default values provided by the original implementations, while all other parameters were kept at their default settings. The clustering performance of each candidate parameter configuration across nine CITE‐seq datasets was evaluated, using metrics such as ARI, NMI, and AMI to quantify performance. Each method's final hyperparameter settings were selected as those that achieved the best average performance across these datasets. The detailed results of these hyperparameter sweep experiments are provided in Figures S24– S31 (Supporting Information), and the optimal configurations identified for each baseline method are summarized in Table S4 (Supporting Information).

### Evaluation Metrics

Adjust Rand Index (*ARI*),^[^ ^36^ ^]^ Normalized Mutual Information (*NMI*),^[^ ^35^ ^]^ Adjusted Mutual Information (*AMI*),^[^ ^37^ ^]^ and Accuracy (*ACC*)^[^ ^38^ ^]^ were used as the evaluation metrics to evaluate the performance of the proposed model. They provide a reliable indication of the similarity between predicted and ground truth labels. The clustering result with the higher value denotes better performance.

*NMI* and *AMI* quantify the mutual information shared between two clustering results, ranging from 0 to 1, *ARI* and *ACC* measure the agreement between two clustering results, ranging from –1 to 1 and from 0 to 1, respectively. In particular, *NMI* focuses on normalizing mutual information to reflect the similarity between the clustering results and true labels, whereas *AMI* emphasizes adjusting mutual information to address random assignment and class imbalance issues, *ARI* measures the similarity between two partitions of the data whereas *ACC* directly compares the alignment of predicted clustering labels with true labels. Given π_*e*_, the predicted label, and π_*t*_, the ground truth label, they can be defined as follows:

(19)

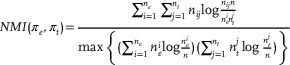



(20)

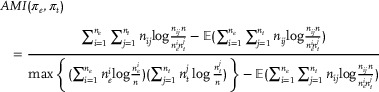



(21)

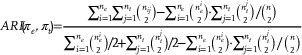

(22) $$ACC\left(\pi_{e},\pi_{t}\right)=\frac{\sum_{i=1}^{n}\delta \left({\pi_{t}}_{i},\mathrm{map}\left({\pi_{e}}_{i}\right)\right)}{n}$$

(23) $$\delta \left(a,b\right)=\left\{\begin{array}{c} 1,\;\;\text{if}\;a=b\\ 0,\;\;\text{otherwise}\; \end{array}\right.$$

where *n*_*e*_, *n*_*t*_ are the cluster numbers in π_*e*_ and π_*t*_, respectively. $n_{e}^{i}$ is the number of samples in cluster *i* of π_*e*_, $n_{t}^{j}$ is the number of samples in cluster *j* of π_*t*_, and *n*_*ij*_ is the intersection sample size between clusters *i* and *j*, E(·) is the expected mutual information between clusters *i* and *j*. *n* is the number of cells in the multi‐omics data, map(·) is a mapping function, usually implemented by the Hungarian algorithm.^[^ ^61^ ^]^ δ(·) is the indicator function.

## Conflict of Interest

The authors declare no conflict of interest.

## Author Contributions

Y.S. and Y.S. contributed equally to this work. Y.S. and Y.W. conceived the study. Y.S., Y.W., and X.L. drafted the manuscript. Y.S., Y.C., and Y.S. collected and analyzed the single‐cell CITE‐seq data. Y.S., Y.S., and Y.W. implemented the scMHVA algorithm. Y.C., X.L., and K.C.W. provided important advice on cell‐type annotation and immune cell dynamics analysis. X.L. supervised the project. All authors wrote the manuscript and read and approved the final manuscript.

## Supporting information

Supporting Information

## Data Availability

We collected twelve real and public CITE‐seq datasets from different platforms. The dataset ‘10x1kpbmc’ can be downloaded from (https://www.10xgenomics.com/datasets/1‐k‐pbm‐cs‐from‐a‐healthy‐donor‐gene‐expression‐and‐cell‐surface‐protein‐3‐standard‐3‐0‐0), the dataset ‘10x5kpbmc’ can be downloaded from (https://support.10xgenomics.com/single‐cell‐gene‐expression/datasets/3.0.2/5k_pbmc_protein_v3) and the dataset ‘10x5kpbmc_TotalSeq’ can be downloaded from (https://www.10xgenomics.com/datasets/5‐k‐peripheral‐blood‐mononuclear‐cells‐pbm‐cs‐from‐a‐healthy‐donor‐with‐cell‐surface‐proteins‐next‐gem‐3‐1‐standard‐3‐1‐0), they are all available on the 10X Genomics website. The ‘GSE100866’, ‘GSE128639’, and ‘GSE194122’ datasets used in our study are accessed in the GEO database by searching GSE100866, GSE128639, and GSE194122, respectively. The ‘inhouse’ dataset and its corresponding cell type labels are obtained from the BREMSC GitHub repository, which can be accessed at https://github.com/tarot0410/BREMSC/tree/master/data. Another dataset ‘10xmalt’ is collected from malt tumor cells and can be downloaded from (https://support.10xgenomics.com/single‐cell‐gene‐expression/datasets/3.0.0/malt_10k_protein_v3). Then, the ‘spleen_lymph’ and ‘PBMC_spector’ datasets and their cell‐type labels can be obtained from the scMDC Github repository at (https://github.com/xianglin226/scMDC/tree/master/datasets). The Haniffa COVID dataset can be downloaded from (https://www.ebi.ac.uk/biostudies/arrayexpress/studies/E‐MTAB‐10026). And the published mouse thymocyte dataset can be downloaded from (https://github.com/YosefLab/Thymus_CITE‐seq). We provide a detailed overview of each dataset in Table S5 (Supporting Information), including the name, data type, species, sample information, the number of cells, gene dimension, ADT dimension, the number of cell types, and the number of batches. All the datasets can be downloaded from (https://zenodo.org/records/16762535). The source code of scMHVA is available at https://github.com/synnnnan/scMHVA.
