# Supplementary material for: Dynamic Synthesis of Multi‐Modal Representations for CITE‐seq Data Integration and Analysis
Source: Adv Sci (Weinh). 2025 Sep 8;12(44):e09247. doi: 10.1002/advs.202509247 (PMC12667526; doi:10.1002/advs.202509247)
Supplement: Supplementary file 1 — Supporting Information [file ADVS-12-e09247-s001.pdf]

Supplementary Information "Dynamic Synthesis of Multi-Modal Representations for CITE-seq Data Integration and Analysis"

Yinan Shi[1†], Yanchi Su[2†], Yue Cheng[1], Ka-Chun Wong[3], Yunhe Wang[4*], and Xiangtao Li[1*]

[1]School of Artificial Intelligence, Jilin University, Changchun, Jilin, China
[2]School of Information Science and Technology, Northeast Normal University, Changchun, Jilin, China
[3]Department of Computer Science, City University of Hong Kong, 83 Tat Chee Ave, Kowloon Tong, Kowloon, Hong Kong SAR
[4]School of Artificial Intelligence, Hebei University of Technology, Tianjin, Tianjin, China
[†]These Authors contributed equally to this work.

Contents

1 scMHVA Exhibits Superior Clustering Accuracy and Computational Efficiency in Multi-omics CITE-seq Data Analysis. 2

2 scMHVA Learns Fine-grained Cellular Interactions through Multi-modal Integration of CITE-seq Data. 8

3 Hyperparameter Evaluation and Ablation Study. 12

4 scMHVA Accurately Identifies Cell Types and Reveals Dynamics of Immune Cell Development through multi-modal CITE-seq analysis. 18

5 Methods. 22

DRAFT

Supplementary Note 1: scMHVA Exhibits Superior Clustering Accuracy and Computational Efficiency in Multi-omics CITE-seq Data Analysis.

To rigorously evaluate the quality of the learned embeddings and ensure the observed performance differences are attributable to the embedding methods themselves rather than the choice of downstream clustering algorithm, we conducted a comprehensive analysis using multiple clustering procedures. Specifically, we applied three different clustering algorithms (k-means, Leiden, and Louvain) to the embeddings produced by scMHVA and all baseline methods. As shown in Supplementary Figure 1-4, scMHVA maintains the highest clustering accuracy regardless of the clustering algorithm, underscoring the robustness and versatility of the scMHVA embeddings. Furthermore, to assess the intrinsic quality of the learned embedding space, we applied k-means, Leiden, and Louvain directly to the scMHVA embedding space. As depicted in Supplementary Figure 1d, scMHVA yields consistently strong and stable clustering performance across all three algorithms. This indicates that the embeddings themselves are of high intrinsic quality and generalize well across different clustering strategies, affirming that scMHVA's superior performance is indeed due to its embedding quality rather than a particular clustering technique.

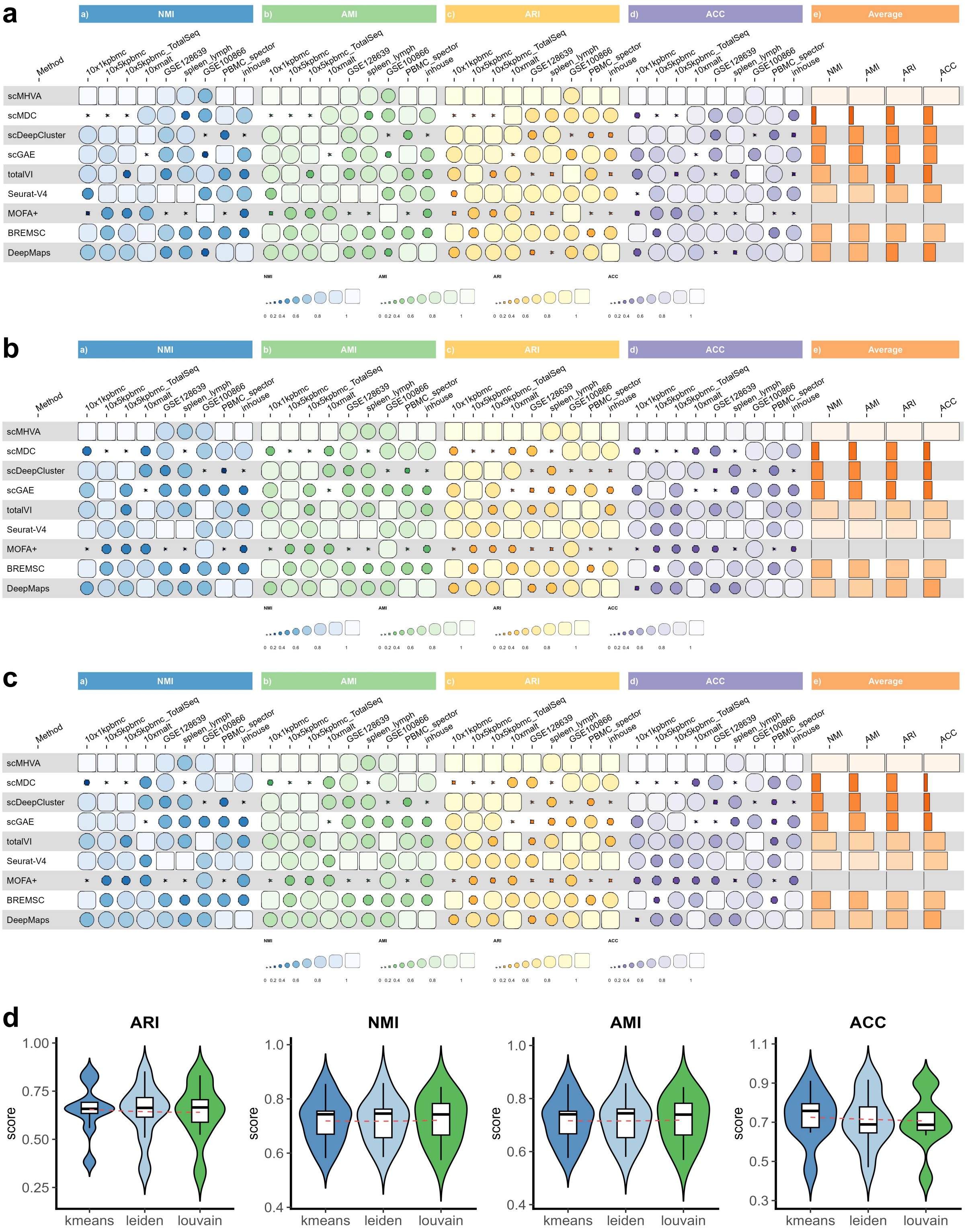

Supplementary Figure 1 a-c Clustering performance of each method on nine datasets using k-means (**a**), Leiden (**b**), and Louvain (**c**), evaluated by NMI, AMI, ARI, and ACC, along with their average scores. **d** Clustering performance of scMHVA was evaluated using k-means, Leiden, and Louvain clustering algorithms.

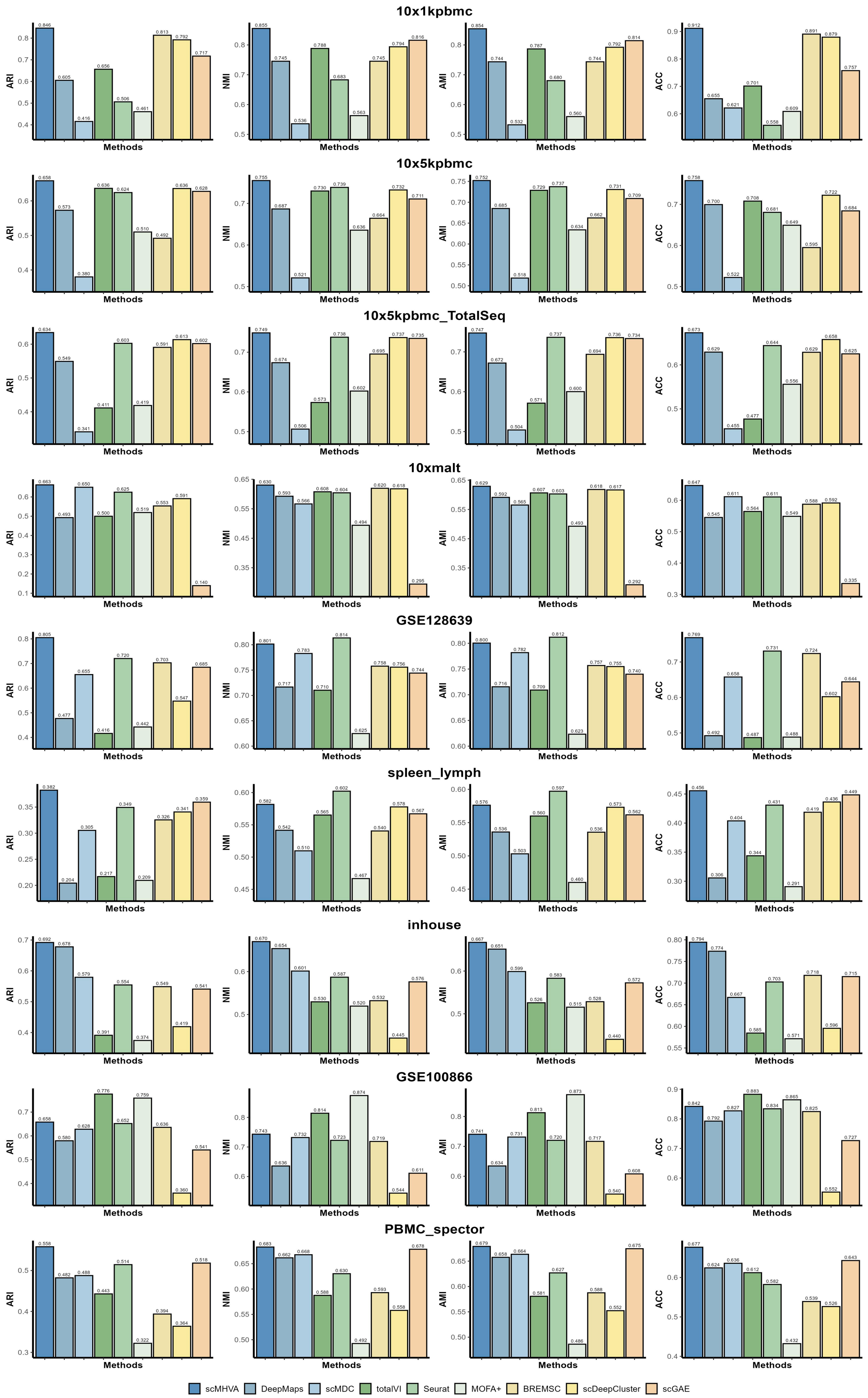

Supplementary Figure 2 Clustering performance of each method on nine datasets using k-means, evaluated by ARI, NMI, AMI, and ACC.

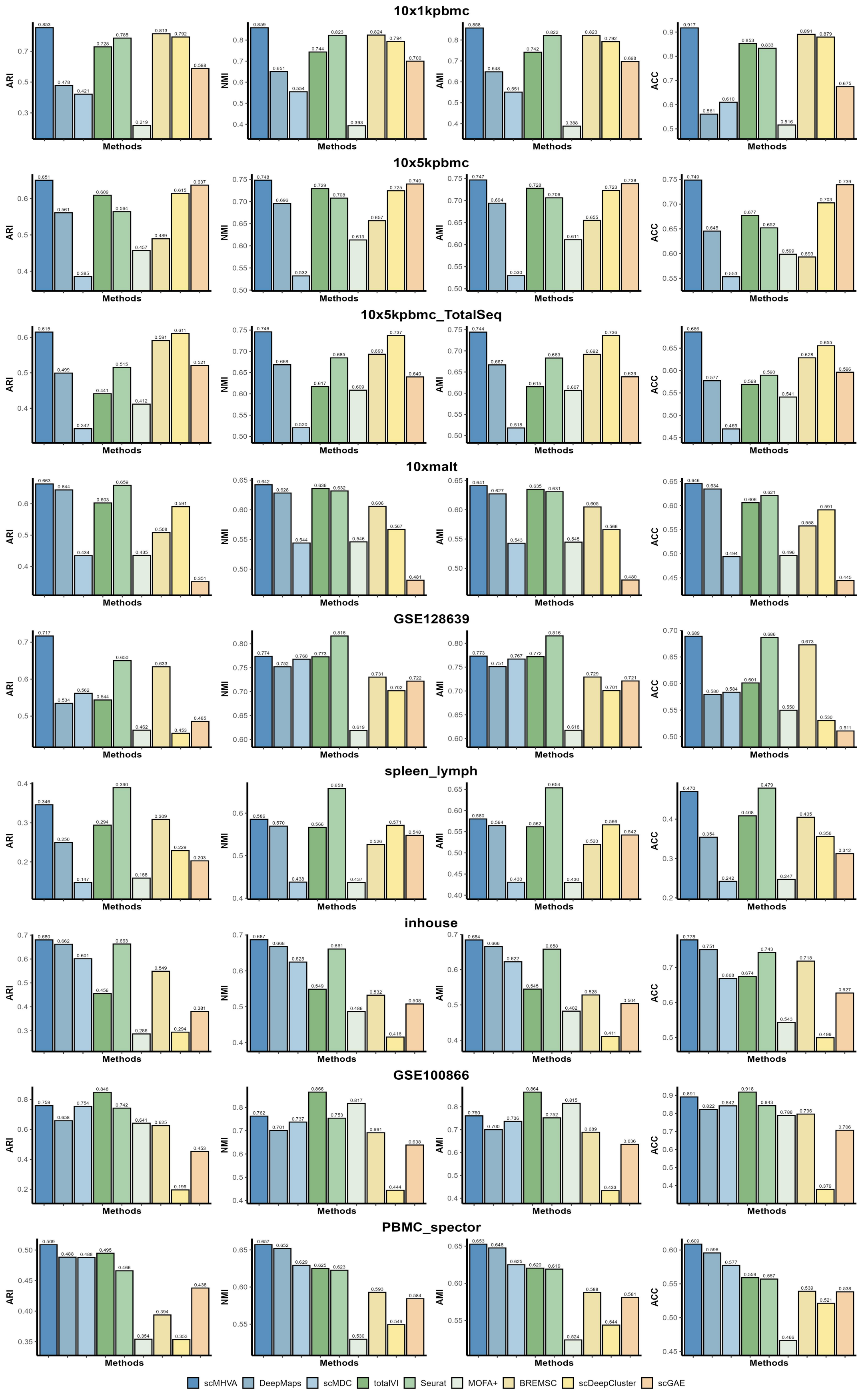

Supplementary Figure 3 Clustering performance of each method on nine datasets using Leiden, evaluated by ARI, NMI, AMI, and ACC.

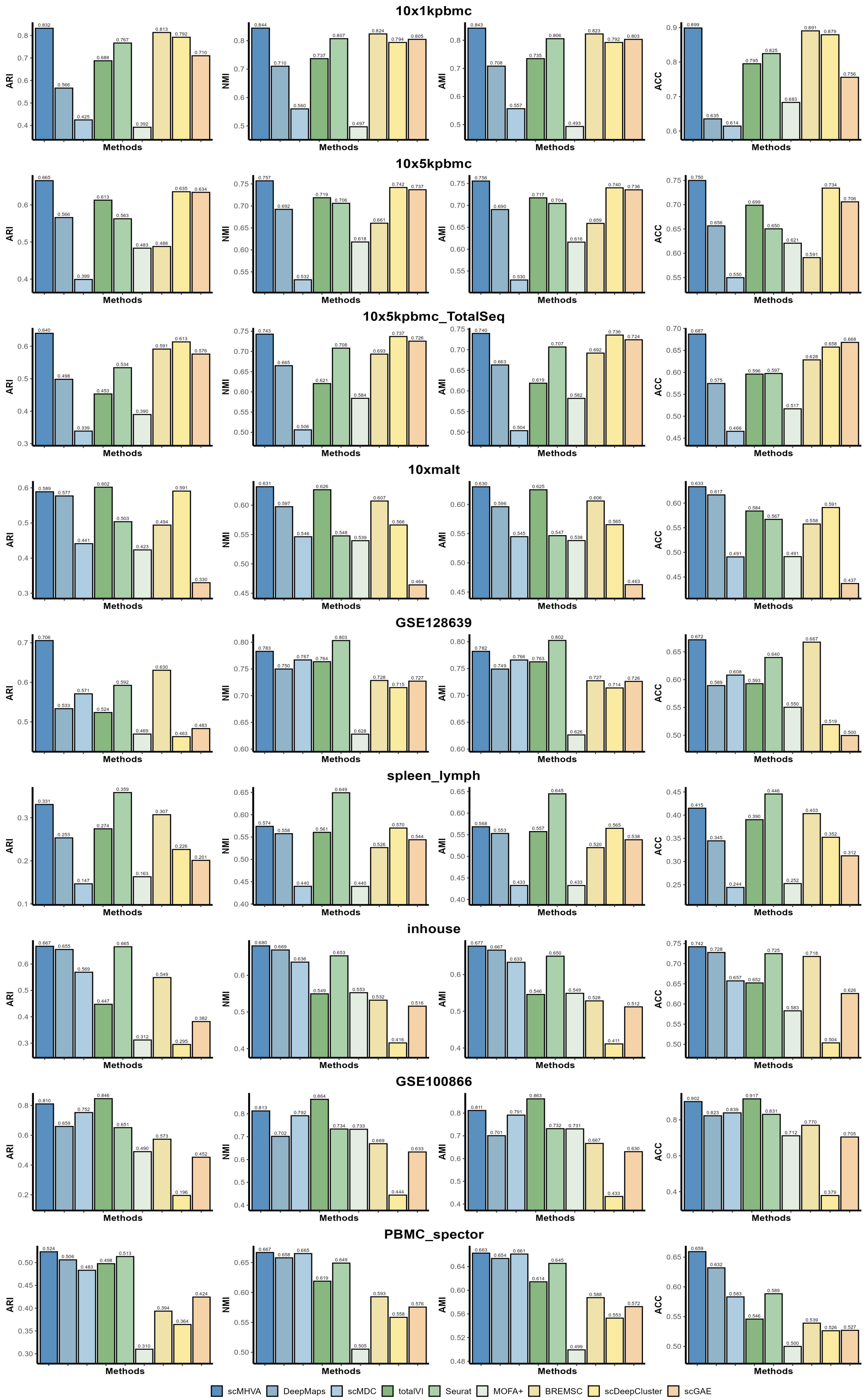

Supplementary Figure 4 Clustering performance of each method on nine datasets using Louvain, evaluated by ARI, NMI, AMI, and ACC.

Method		scMHVA	scMDC	scDeepCluster	scGAE	totalVI	DeepMaps	Seurat	MOFA+	BREMSC
Time(s)	10x1kpbmc	1.51E+01	5.00E+01	3.51E+03	2.75E+02	8.47E+02	3.66E+02	1.80E+01	5.07E+03	7.88E+02
	10x5kpbmc	7.30E+01	3.73E+02	2.48E+04	4.17E+02	2.99E+03	7.55E+02	5.60E+01	3.84E+04	1.22E+05
	10x5kpbmc_TotalSeq	8.69E+01	6.03E+02	2.08E+04	5.40E+02	2.23E+03	7.35E+02	6.30E+01	4.06E+04	1.86E+05
	10xmalt	1.60E+02	6.46E+02	3.29E+04	9.29E+02	2.34E+03	1.12E+03	1.04E+02	5.10E+04	2.89E+05
	GSE128639	4.47E+02	1.99E+03	6.19E+04	3.98E+03	4.60E+03	3.39E+03	3.77E+02	1.44E+05	5.53E+05
	spleen_lymph	3.01E+02	9.02E+02	2.52E+04	1.92E+03	4.61E+03	2.60E+03	1.58E+02	6.87E+04	3.27E+05
	inhouse	2.27E+01	9.70E+01	4.65E+03	2.37E+02	4.66E+03	2.10E+02	2.90E+01	1.01E+04	1.87E+03
	GSE100866	1.82E+01	8.50E+01	5.37E+02	2.26E+02	3.28E+03	7.52E+02	8.10E+01	9.28E+03	1.56E+03
	PBMC_spector	5.35E+01	2.75E+02	1.30E+04	2.71E+02	3.88E+03	5.40E+02	1.27E+02	2.56E+04	1.07E+05
Parameter (M)	10x1kpbmc	0.8092	17.9828	17.9936	1.7484	35.9148	0.9942	/	/	/
	10x5kpbmc	0.8144	21.4517	21.473	1.7579	35.9392	5.6416	/	/	/
	10x5kpbmc_TotalSeq	0.8144	22.0484	22.0698	1.7579	35.9392	5.9286	/	/	/
	10xmalt	0.8099	22.6813	22.6921	1.7484	35.9148	8.8857	/	/	/
	GSE128639	0.8123	17.515	17.5314	1.7535	18.2914	31.7022	/	/	/
	spleen_lymph	0.8386	13.9934	14.0715	1.8086	14.7451	17.5121	/	/	/
	inhouse	0.8078	17.2884	17.2942	1.744	35.9035	1.6697	/	/	/
	GSE100866	0.8045	16.6825	16.6883	1.744	35.8779	1.475	/	/	/
	PBMC_spector	0.8196	19.2357	19.2691	1.7687	35.9668	4.1195	/	/	/
Encoder	Depth	3	4	3	2	3	3	/	/	/
	Layers	[200, 100, 100]	[256, 64, 32, 16]	[256, 64, 32]	[120, 25]	[256, 256, 20]	[512, 256, 128]	/	/	/
Decoder	Depth	1	3/2	3	4	2	3	/	/	/
	Layers	[100]	[16, 64, 256]/[16, 20]	[32, 64, 256]	[25, 64, 256, 512]	[20, 256]	[128, 256, 512]	/	/	/

Supplementary Table 1 Comparison of runtime, model complexity (number of parameters), and architecture details (encoder/decoder depth and layer dimensions) of scMHVA and baseline methods across multiple CITE-seq datasets. All runtimes are reported in seconds, and parameter counts are in millions (M).

Module	Dataset	Time(s)	Percentage (%)
multi-head attention	10x1kpbmc	2.6	17
	10x5kpbmc	10.5	16.5
	10x5kpbmc_TotalSeq	11.2	16.6
	10xmalt	26.4	16.5
	GSE128639	74.2	16.6
	spleen_lymph	49.6	16.5
	inhouse	3.7	16.4
	GSE100866	3	16.4
	PBMC_spector	7.6	16.8
adaptive fusion	10x1kpbmc	1	6.6
	10x5kpbmc	4.5	7.1
	10x5kpbmc_TotalSeq	4.7	7
	10xmalt	10.4	6.5
	GSE128639	25.9	5.8
	spleen_lymph	19.5	6.5
	inhouse	1.5	6.4
	GSE100866	1.2	6.5
	PBMC_spector	3	6.7

Supplementary Table 2 The running time and proportion of the multi-head attention mechanism and adaptive fusion module during training.

Supplementary Note 2: scMHVA Learns Fine-grained Cellular Interactions through Multi-modal Integration of CITE-seq Data.

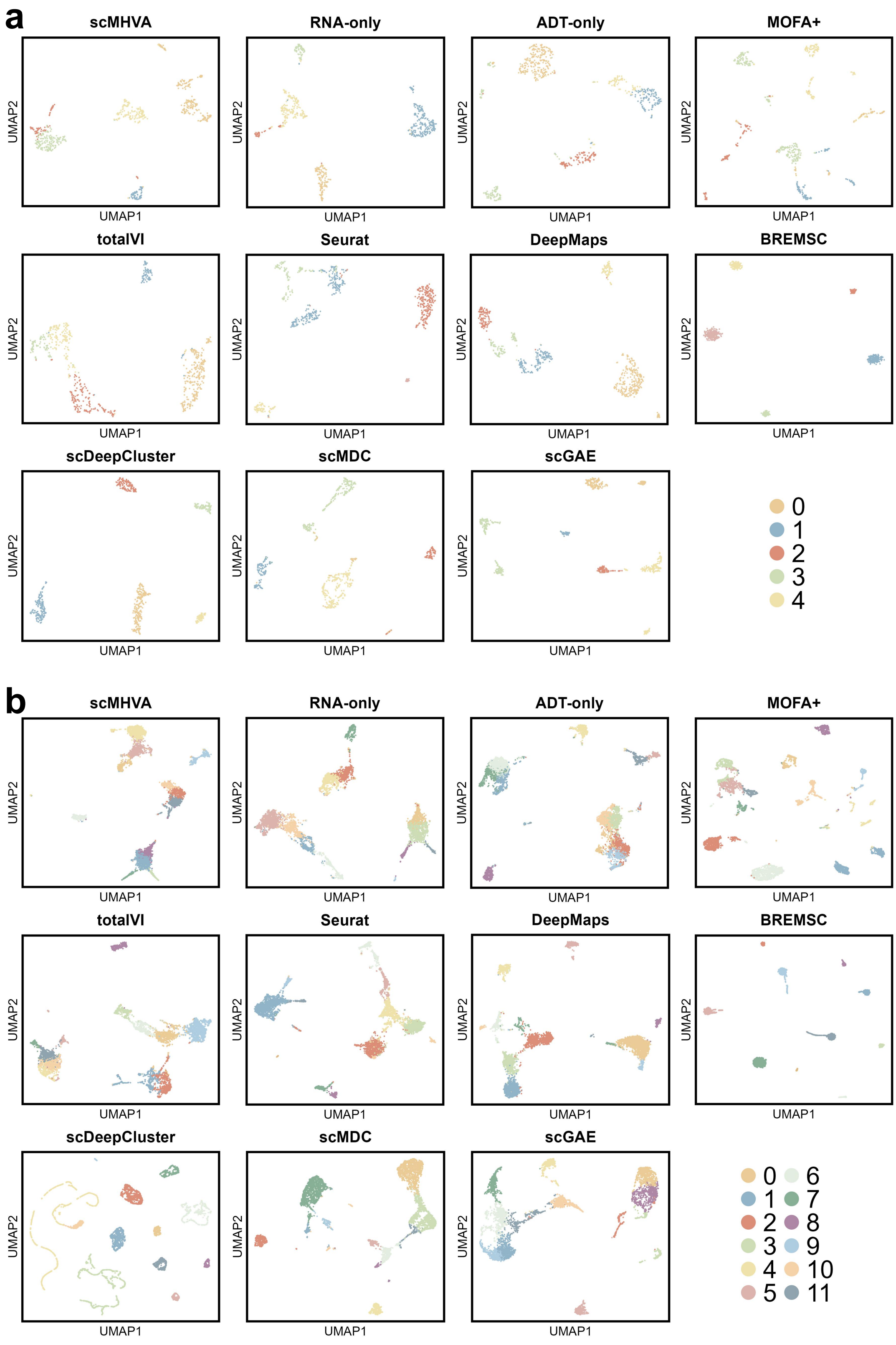

Supplementary Figure 5 UMAP visualizations of the cell embeddings for scMHVA and competing methods. **a** Low-dimension representation of scMHVA and other competing methods on the '10x1kpbmc' dataset. **b** Low-dimension representation of scMHVA and other competing methods on the '10x5kpbmc' dataset.

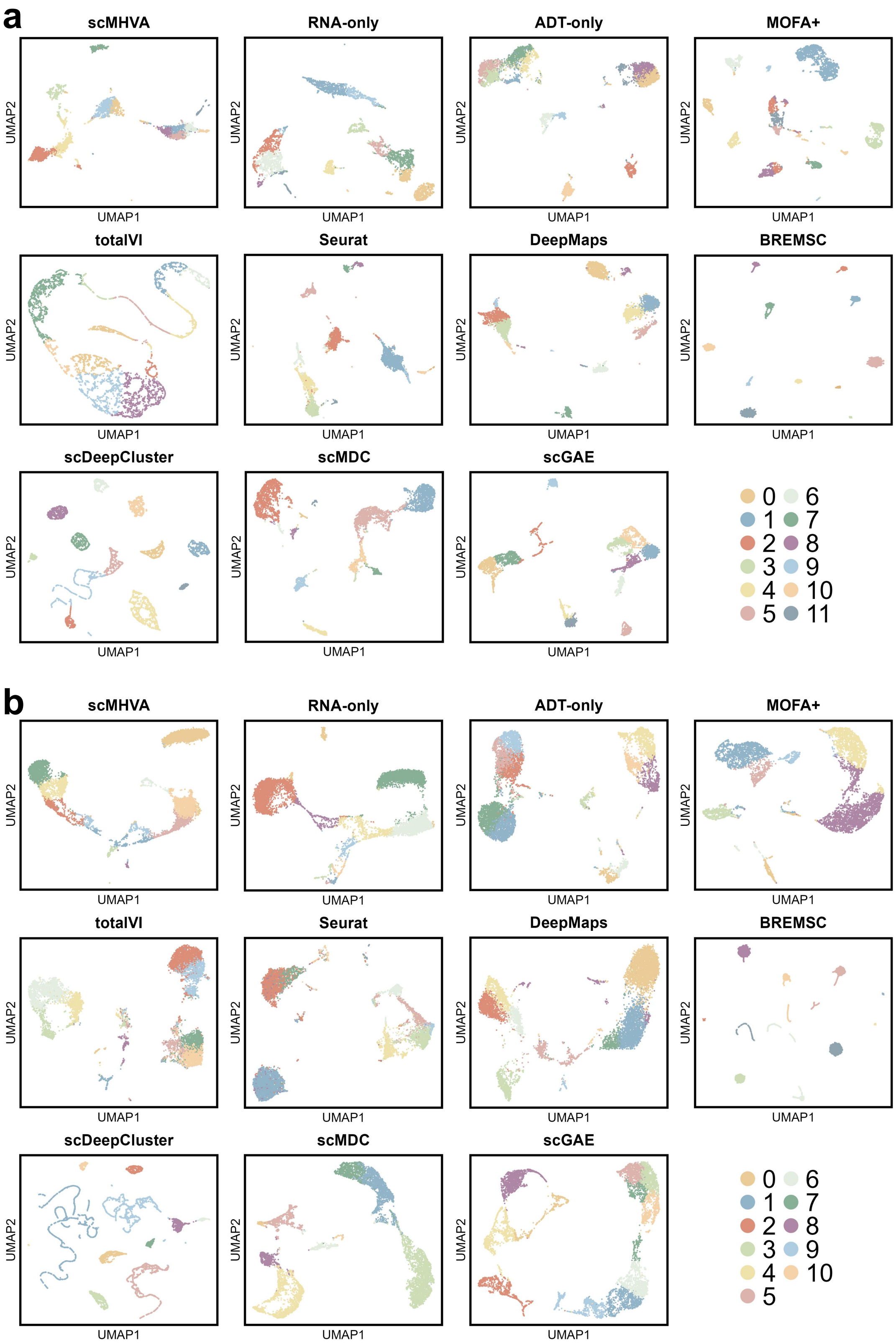

Supplementary Figure 6 UMAP visualizations of the cell embeddings for scMHVA and competing methods. **a** Low-dimension representation of scMHVA and other competing methods on the '10x5kpbmc_TotalSeq' dataset. **b** Low-dimension representation of scMHVA and other competing methods on the '10xmalt' dataset.

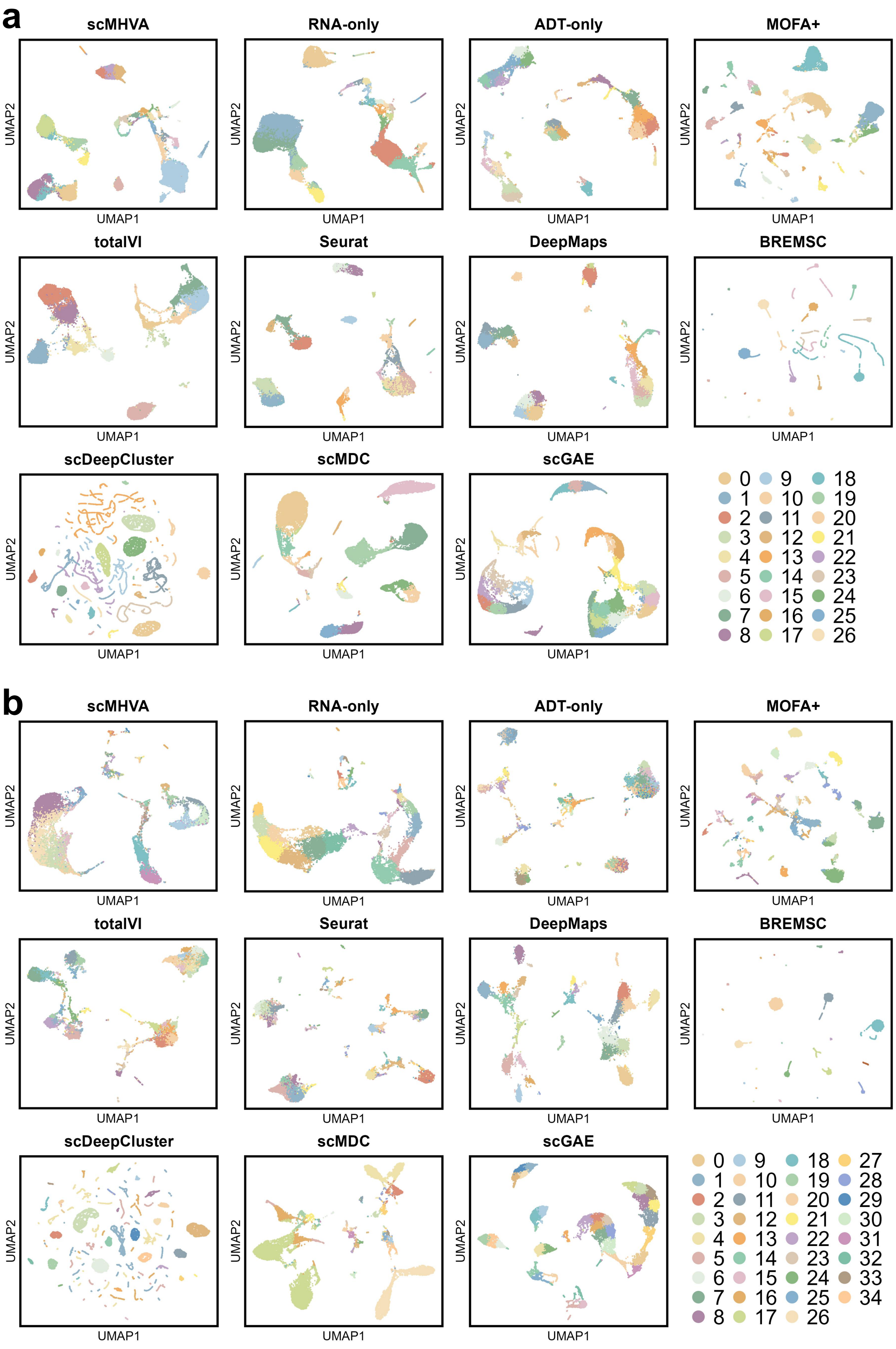

Supplementary Figure 7 UMAP visualizations of the cell embeddings for scMHVA and competing methods. **a** Low-dimension representation of scMHVA and other competing methods on the 'GSE128639' dataset. **b** Low-dimension representation of scMHVA and other competing methods on the 'spleen_lymph' dataset.

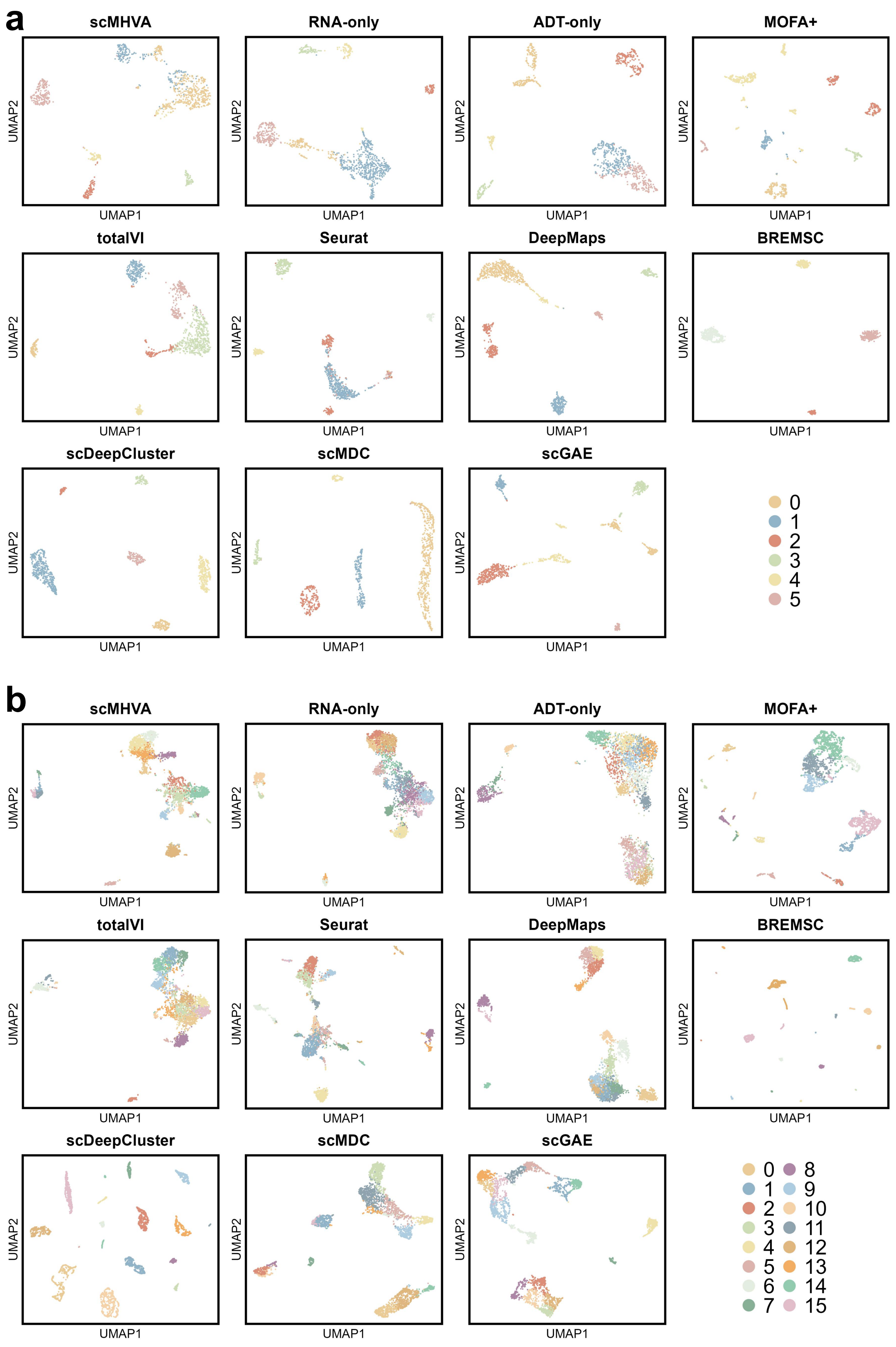

Supplementary Figure 8 UMAP visualizations of the cell embeddings for scMHVA and competing methods. **a** Low-dimension representation of scMHVA and other competing methods on the 'GSE100866' dataset. **b** Low-dimension representation of scMHVA and other competing methods on the 'PBMC_spector' dataset.

Supplementary Note 3: Hyperparameter Evaluation and Ablation Study.

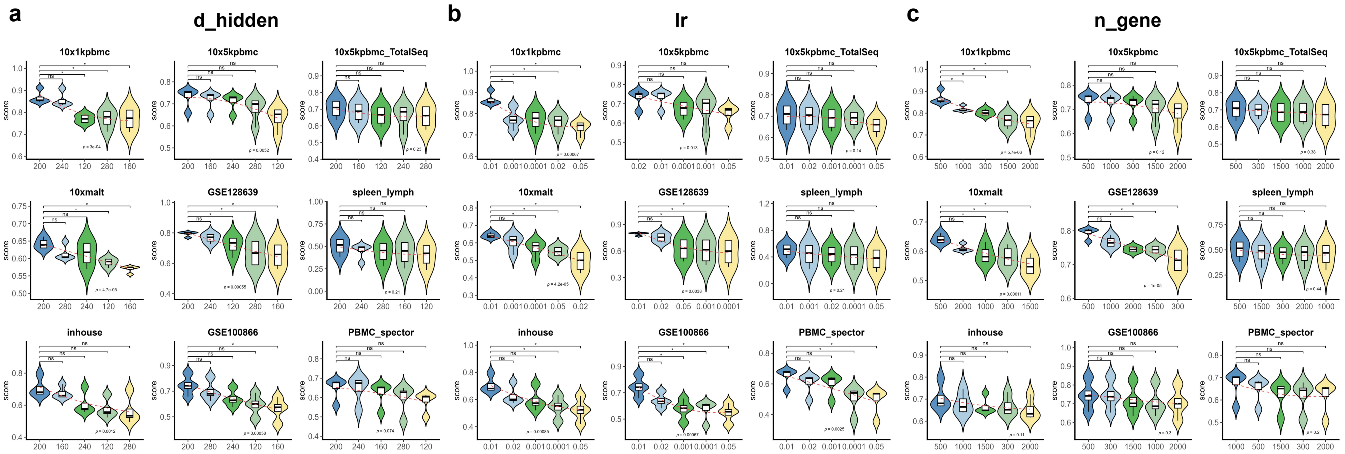

Supplementary Figure 9 Hyperparameter tuning and ablation study of scMHVA. Effect of different learning rates (lr) (**a**), numbers of neurons in the hidden layer (d_hidden) (**b**), and numbers of attention heads (n_head) (**c**) on the clustering performance of CITE-seq datasets.

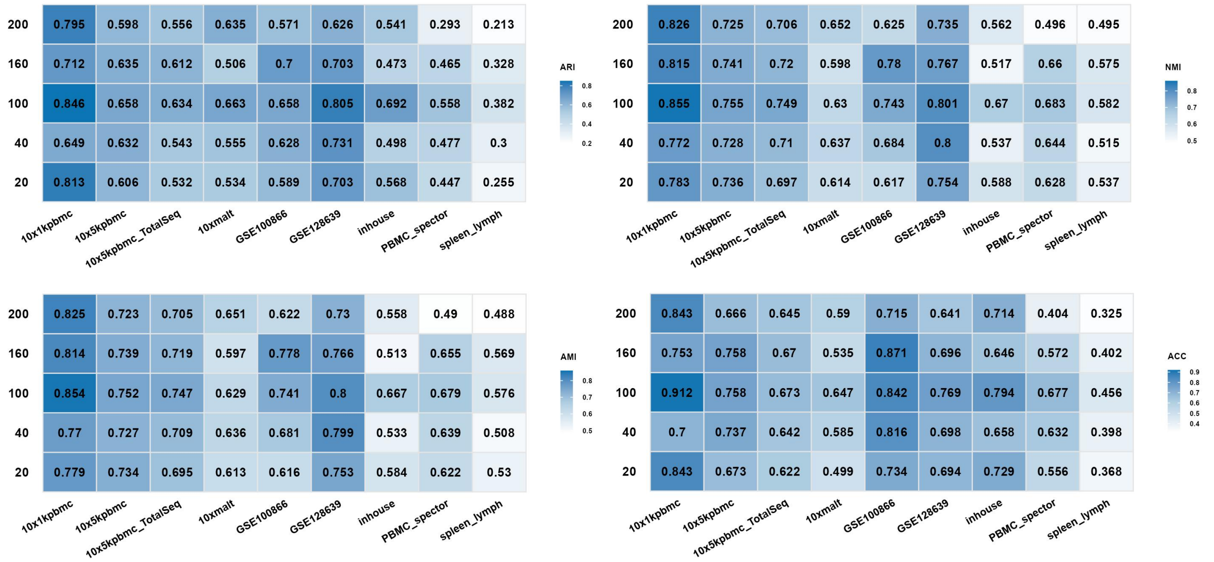

Supplementary Figure 10 Effect of different dimensions of the embedding representation (z_dim) on the clustering performance of CITE-seq datasets.

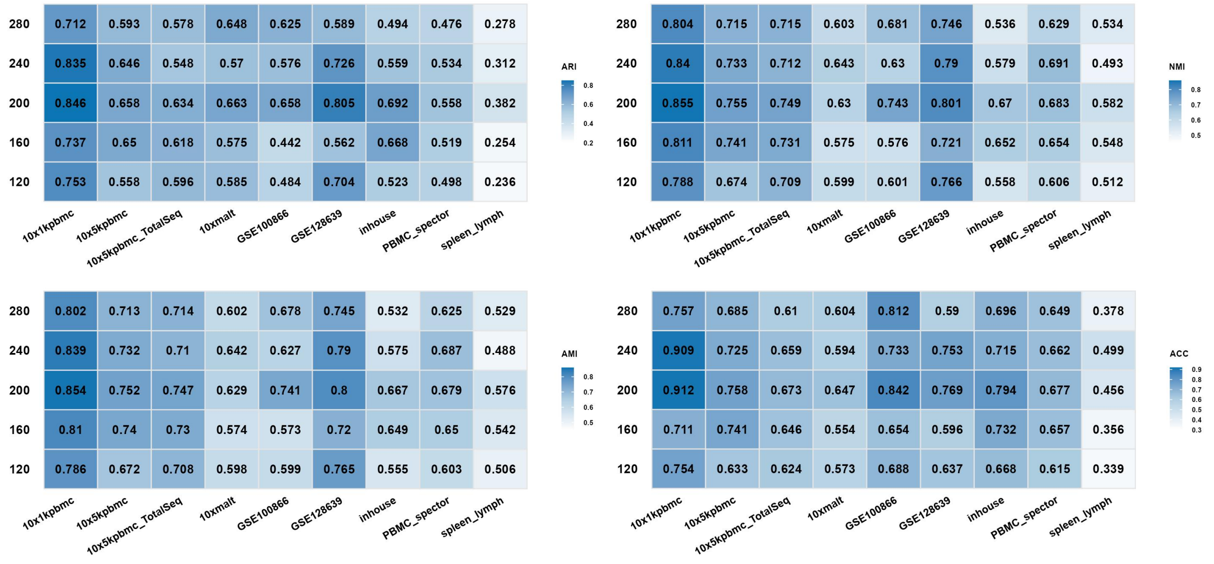

Supplementary Figure 11 Effect of different numbers of neurons in the hidden layer (d_hidden) on the clustering performance of CITE-seq datasets.

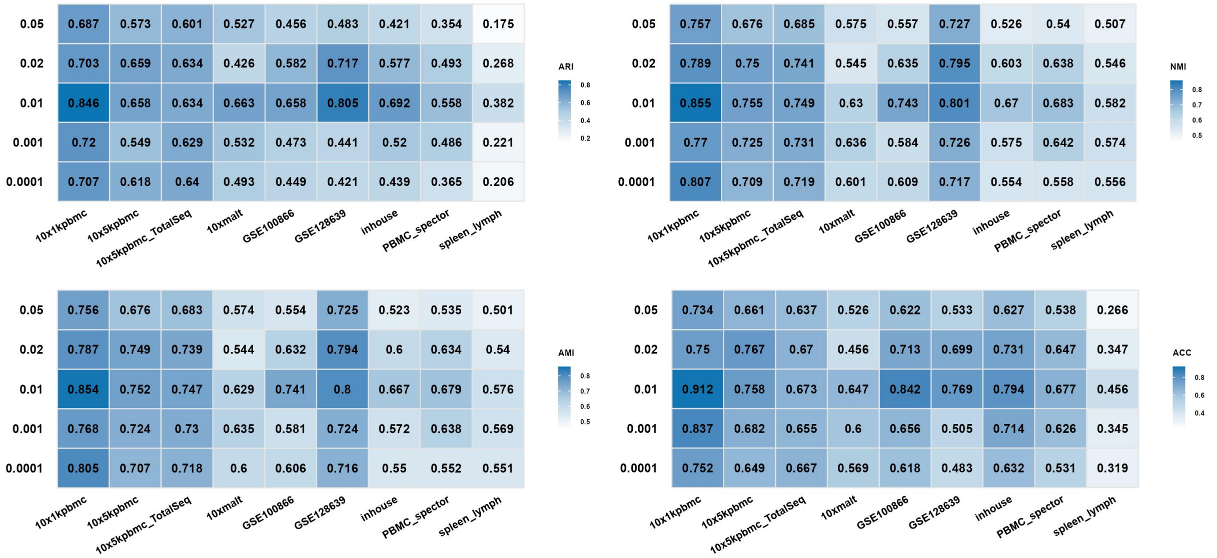

Supplementary Figure 12 Effect of different learning rates (lr) on the clustering performance of CITE-seq datasets.

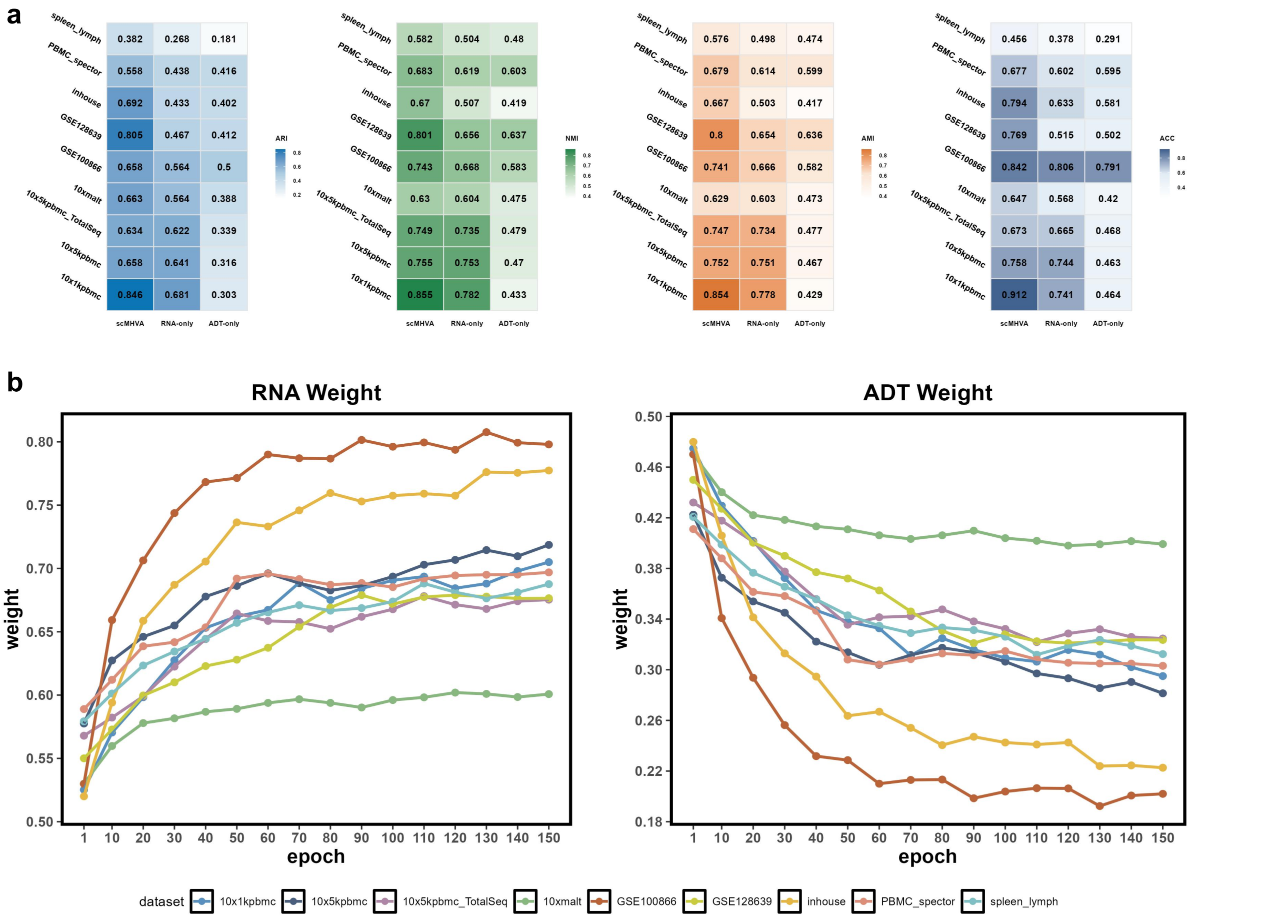

Supplementary Figure 13 a Comparison of clustering performance between RNA-only, ADT-only, and fused modalities using ARI, NMI, AMI, and ACC. **b** The weight changes of RNA and ADT modality during training across different datasets.

Metric	Dataset	dynamic	0.9	0.8	0.7	0.6	0.5	0.4	0.3	0.2	0.1
ARI	10x1kpbmc	**0.8462**	0.8226	0.81	0.8246	0.8222	0.8203	0.8012	0.8065	0.8056	0.8126
	10x5kpbmc	**0.6582**	0.6311	0.6342	0.6476	0.6502	0.6373	0.639	0.6285	0.6203	0.6259
	10x5kpbmc_TotalSeq	**0.6344**	0.617	0.6188	0.624	0.6309	0.5913	0.6256	0.628	0.6257	0.6154
	10xmalt	0.6627	0.6375	0.6467	0.6527	**0.6631**	0.648	0.6409	0.6447	0.629	0.5786
	GSE128639	**0.8051**	0.6955	0.6268	0.789	0.6861	0.6747	0.6573	0.5469	0.5655	0.5567
	spleen_lymph	**0.3824**	0.346	0.3651	0.368	0.3817	0.3806	0.3707	0.3687	0.3677	0.2947
	inhouse	**0.692**	0.6707	0.6358	0.6494	0.5682	0.5409	0.4979	0.6231	0.6183	0.6093
	GSE100866	**0.658**	0.6401	0.6572	0.6442	0.6236	0.603	0.6135	0.5849	0.5955	0.6036
	PBMC_spector	**0.5581**	0.5159	0.5386	0.5406	0.4232	0.4891	0.5143	0.4752	0.4603	0.489
	Average	**0.6552**	0.6196	0.6148	0.6378	0.6055	0.5984	0.5956	0.5896	0.5875	0.5762
NMI	10x1kpbmc	**0.8553**	0.7868	0.8152	0.8368	0.8245	0.8013	0.7968	0.7945	0.8011	0.7993
	10x5kpbmc	**0.755**	0.7256	0.7214	0.7489	0.7446	0.7334	0.7262	0.7112	0.7133	0.7229
	10x5kpbmc_TotalSeq	**0.7487**	0.7262	0.7323	0.7374	0.7428	0.7202	0.7215	0.727	0.7127	0.7059
	10xmalt	0.6304	0.6009	0.6223	0.6248	**0.632**	0.6198	0.6206	0.6234	0.6166	0.5947
	GSE128639	**0.8014**	0.7638	0.7482	0.7888	0.7719	0.7809	0.7622	0.7187	0.7281	0.7181
	spleen_lymph	**0.5817**	0.5445	0.5435	0.5506	0.5769	0.5749	0.5229	0.5237	0.5122	0.5067
	inhouse	**0.6699**	0.626	0.6184	0.6362	0.5734	0.568	0.5776	0.5897	0.5327	0.5448
	GSE100866	**0.7433**	0.7016	0.723	0.7276	0.7056	0.7089	0.6963	0.676	0.6661	0.6627
	PBMC_spector	**0.6829**	0.6475	0.6751	0.6673	0.6131	0.6472	0.6579	0.6266	0.6256	0.6243
	Average	**0.7187**	0.6803	0.6888	0.702	0.6872	0.6838	0.6758	0.6656	0.6565	0.6533
AMI	10x1kpbmc	**0.8542**	0.7852	0.8137	0.8354	0.823	0.7998	0.7953	0.7932	0.7995	0.7978
	10x5kpbmc	**0.7521**	0.7243	0.7201	0.7476	0.7433	0.7321	0.7249	0.7099	0.7119	0.7216
	10x5kpbmc_TotalSeq	**0.7474**	0.725	0.731	0.7361	0.7416	0.7188	0.7203	0.7257	0.7115	0.7047
	10xmalt	0.6293	0.5998	0.6213	0.6237	**0.6308**	0.6188	0.6196	0.6223	0.6156	0.5936
	GSE128639	**0.8005**	0.7628	0.7471	0.788	0.771	0.7801	0.7613	0.7176	0.727	0.7173
	spleen_lymph	**0.5762**	0.5389	0.5381	0.5493	0.5714	0.5695	0.5192	0.5182	0.5066	0.5009
	inhouse	**0.6671**	0.6225	0.6148	0.6327	0.5691	0.5645	0.5737	0.5861	0.5286	0.541
	GSE100866	**0.7405**	0.6993	0.7206	0.7253	0.7034	0.7067	0.694	0.6736	0.6638	0.6603
	PBMC_spector	**0.6791**	0.6432	0.6714	0.6634	0.6084	0.6429	0.6542	0.6228	0.6218	0.6202
	Average	**0.7163**	0.6779	0.6865	0.7002	0.6847	0.6815	0.6736	0.6633	0.654	0.6508
ACC	10x1kpbmc	**0.9116**	0.8633	0.8717	0.8931	0.8862	0.8689	0.8675	0.8664	0.8717	0.8759
	10x5kpbmc	**0.7579**	0.7308	0.7114	0.7517	0.7498	0.7453	0.7323	0.7231	0.725	0.7216
	10x5kpbmc_TotalSeq	**0.6731**	0.6547	0.6661	0.6611	0.6678	0.6142	0.6455	0.6518	0.6502	0.6381
	10xmalt	**0.6472**	0.5984	0.6302	0.6361	0.6463	0.6126	0.6164	0.6141	0.5957	0.5764
	GSE128639	**0.7686**	0.6427	0.5946	0.7398	0.6407	0.6433	0.6151	0.5453	0.5636	0.5575
	spleen_lymph	**0.4556**	0.3734	0.3789	0.3668	0.3966	0.4185	0.4311	0.387	0.3927	0.3983
	inhouse	**0.7945**	0.751	0.773	0.7816	0.7111	0.6457	0.7118	0.7008	0.6957	0.6831
	GSE100866	**0.8422**	0.8282	0.8197	0.836	0.8317	0.8274	0.8208	0.8155	0.8113	0.821
	PBMC_spector	**0.6768**	0.6491	0.6647	0.6636	0.6699	0.6395	0.6416	0.6068	0.6083	0.6124
	Average	**0.7253**	0.6768	0.6789	0.7033	0.6889	0.6684	0.6758	0.6568	0.6571	0.6538

Supplementary Table 3 Clustering performance of nine datasets under static fusion weights.

To better demonstrate the adaptability of our fusion mechanism, we conducted an additional experiment simulating scenarios where the ADT modality was partially degraded. Specifically, we introduced controlled masking of ADT features at varying rates (from 10% to 90%) and measured the clustering performance of scMHVA and the baseline models under each masking condition. As shown in Supplementary Figure 14, scMHVA's performance degraded more slowly than that of the baseline models, indicating superior robustness to modality-specific information loss. Moreover, the learned fusion weights (Supplementary Figure 15) showed that scMHVA automatically down-weighted the degraded ADT modality while correspondingly up-weighting the RNA modality. This dynamic adjustment confirms the model's ability to assess and respond to changes in modality quality or importance, thereby maintaining robust integration and downstream accuracy.

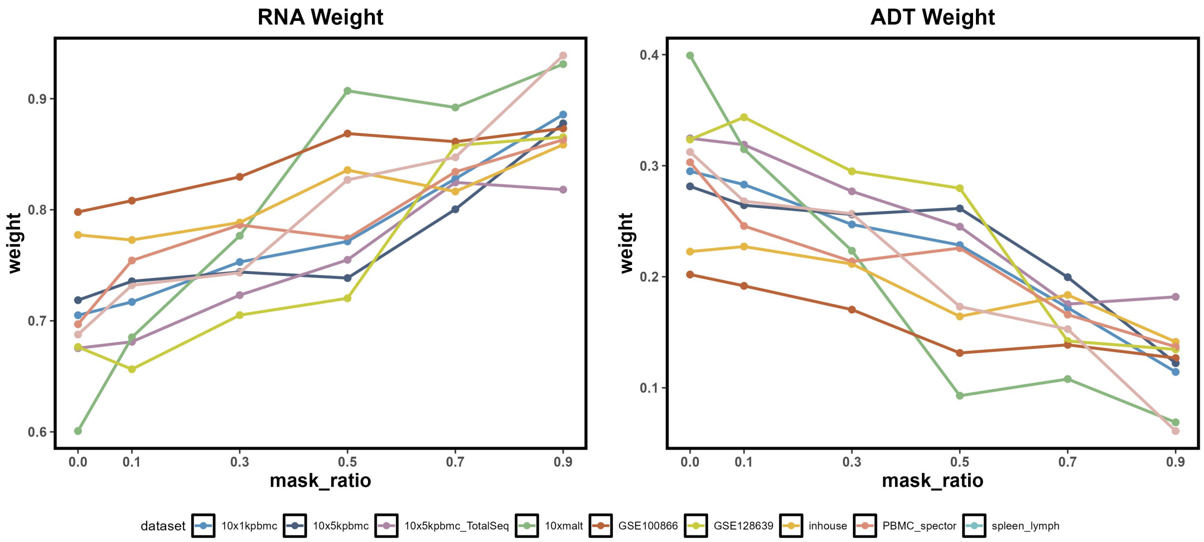

Supplementary Figure 14 The weight changes of RNA and ADT modality across datasets at ADT masking ratios ranging from 0.1 to 0.9.

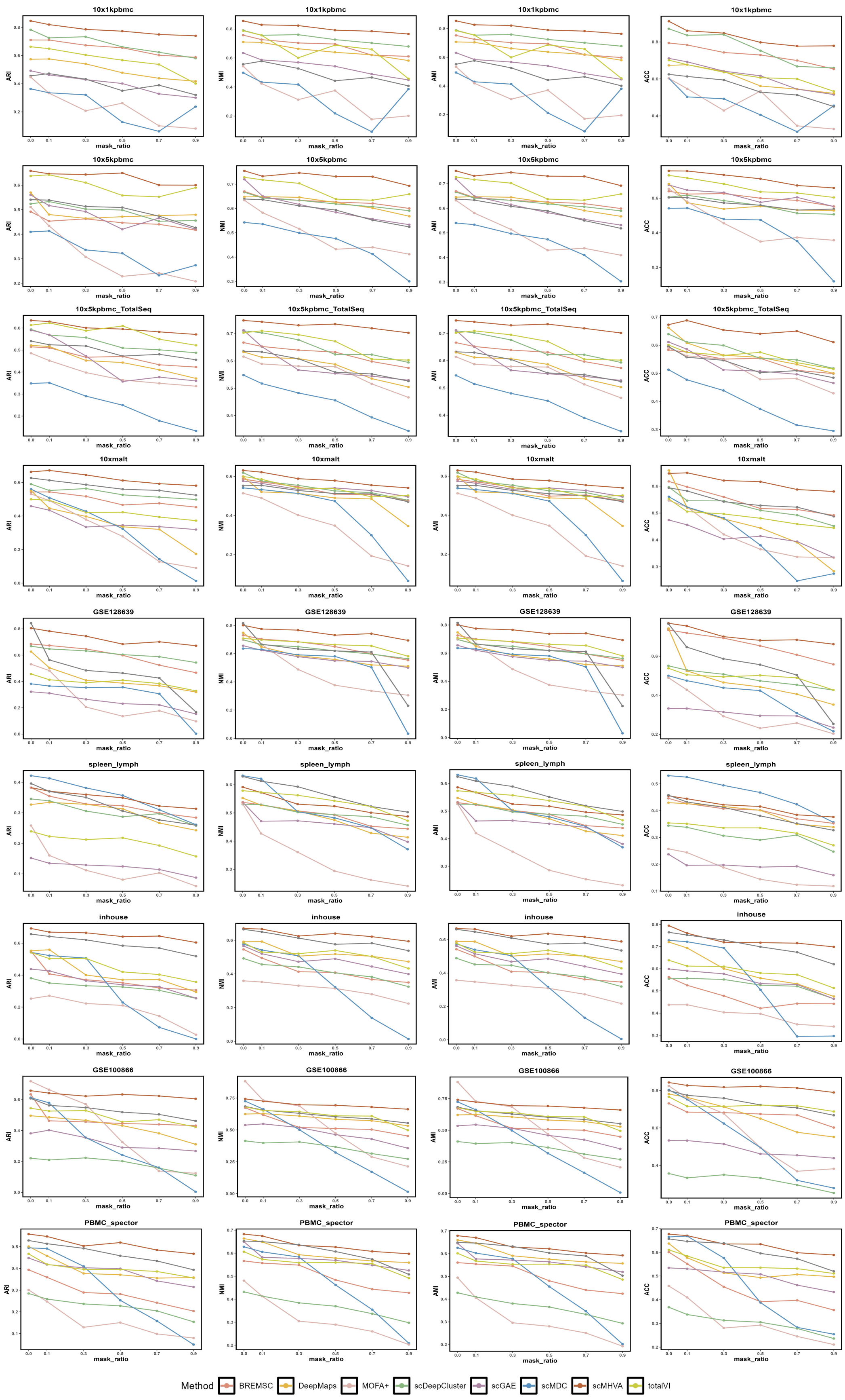

Supplementary Figure 15 Clustering performance of each method across varying ADT mask ratios from 0.1 to 0.9.

Supplementary Note 4: scMHVA Accurately Identifies Cell Types and Reveals Dynamics of Immune Cell Development through multi-modal CITE-seq analysis.

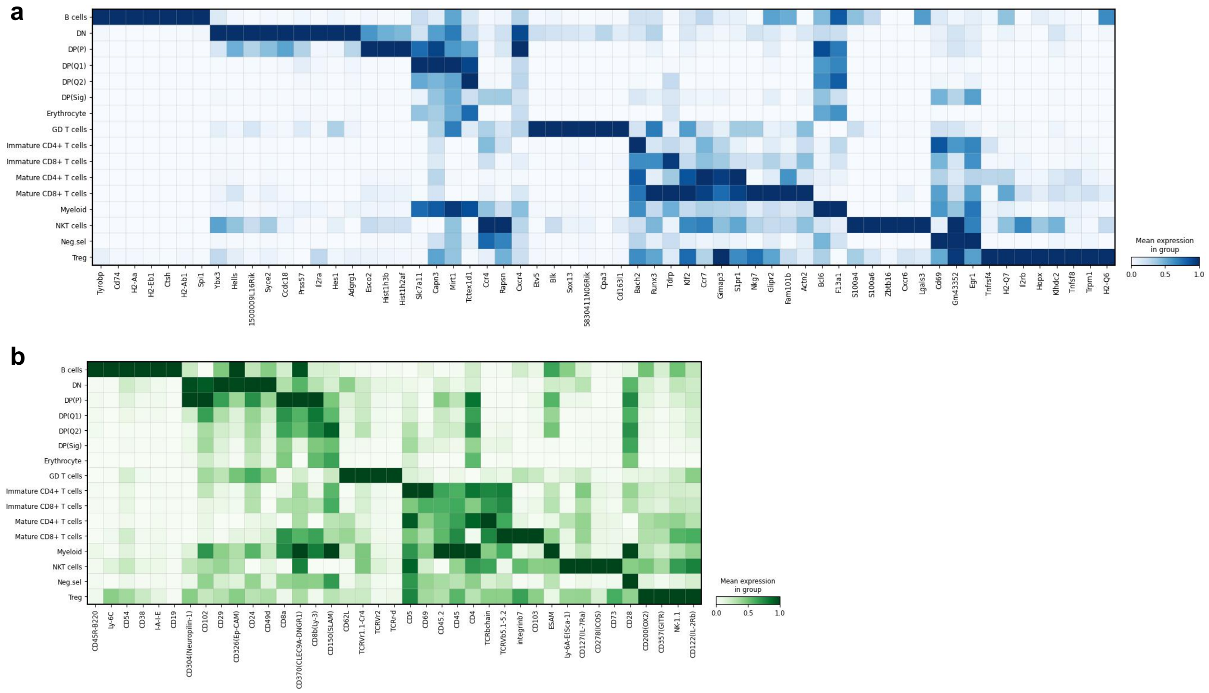

Supplementary Figure 16 Heatmap of marker RNA (a) and marker ADT (b) expression levels for each cell cluster identified by the integrated clustering of RNA and ADT data.

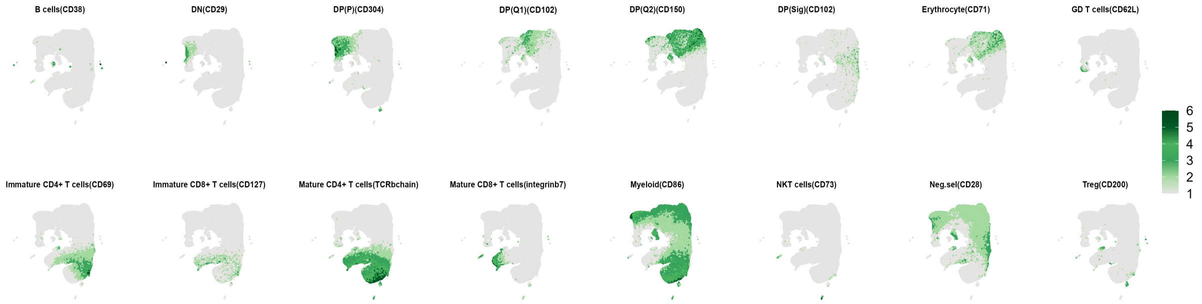

Supplementary Figure 17 Feature plot of marker ADT expression levels for each cell cluster identified by scMHVA integrated clustering.

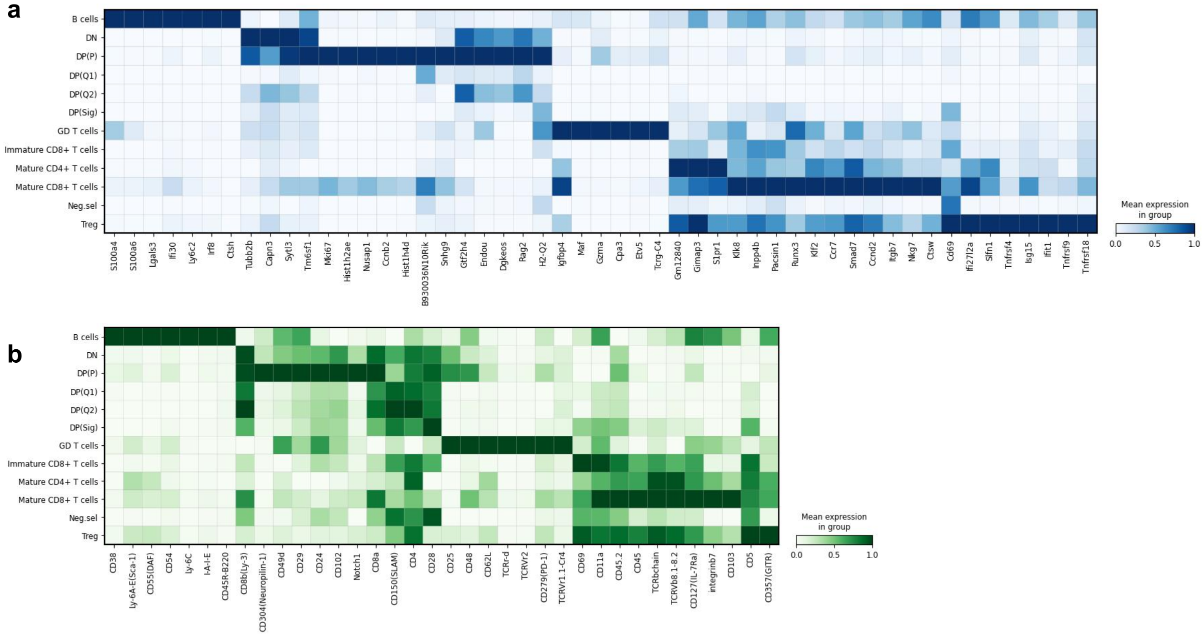

Supplementary Figure 18 Heatmap of marker RNA (a) and marker ADT (b) expression levels for each cell cluster identified by RNA-only clustering.

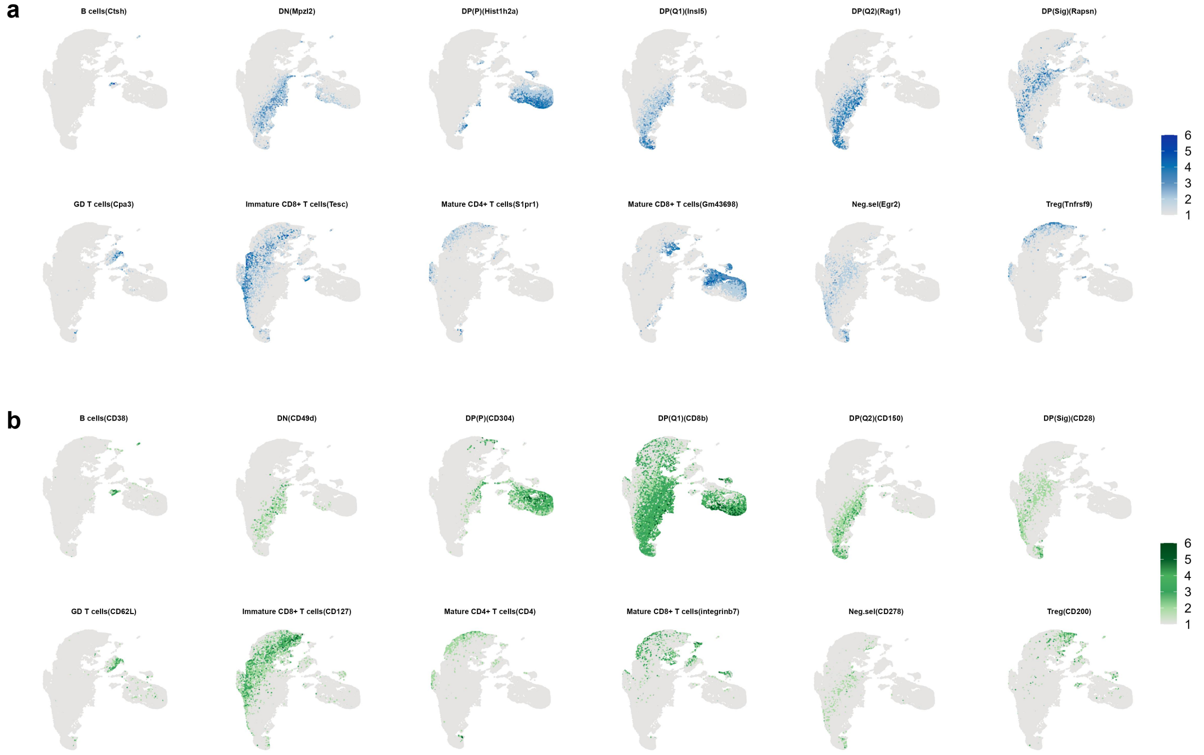

Supplementary Figure 19 Feature plot of marker RNA (a) and marker ADT (b) expression levels for each cell cluster identified by RNA-only clustering.

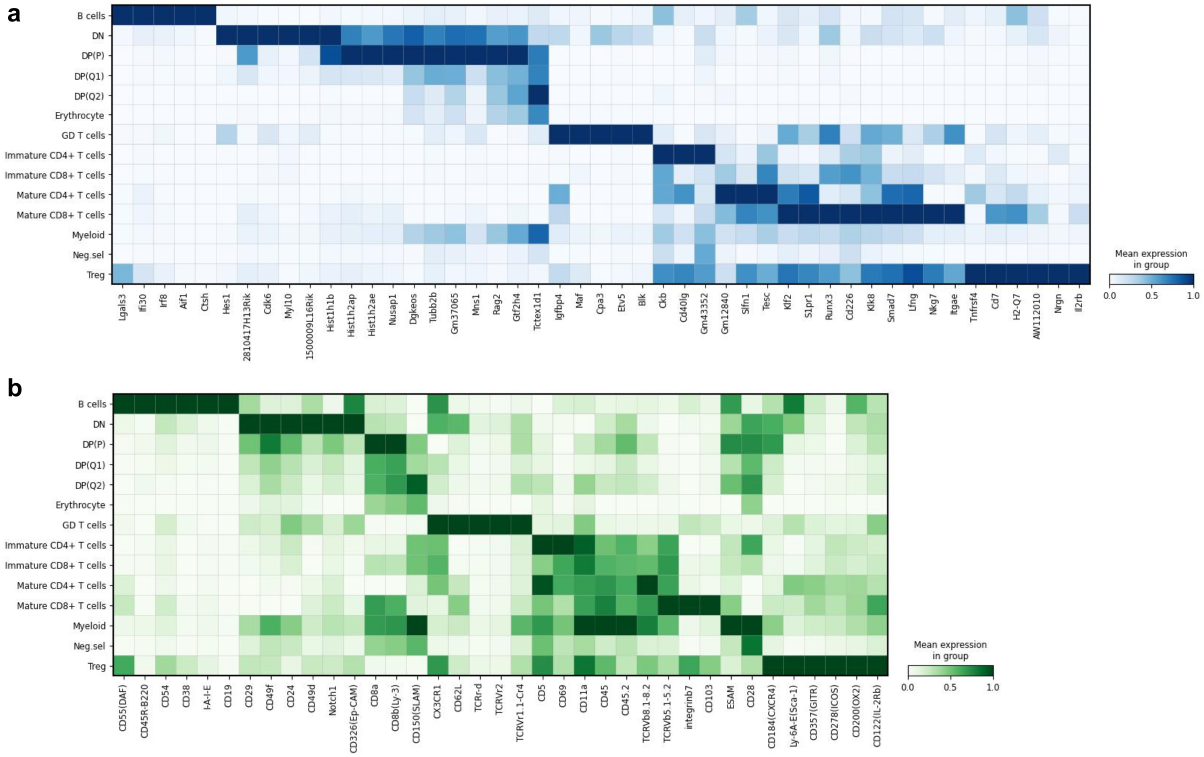

Supplementary Figure 20 Heatmap of marker RNA (a) and marker ADT (b) expression levels for each cell cluster identified by ADT-only clustering.

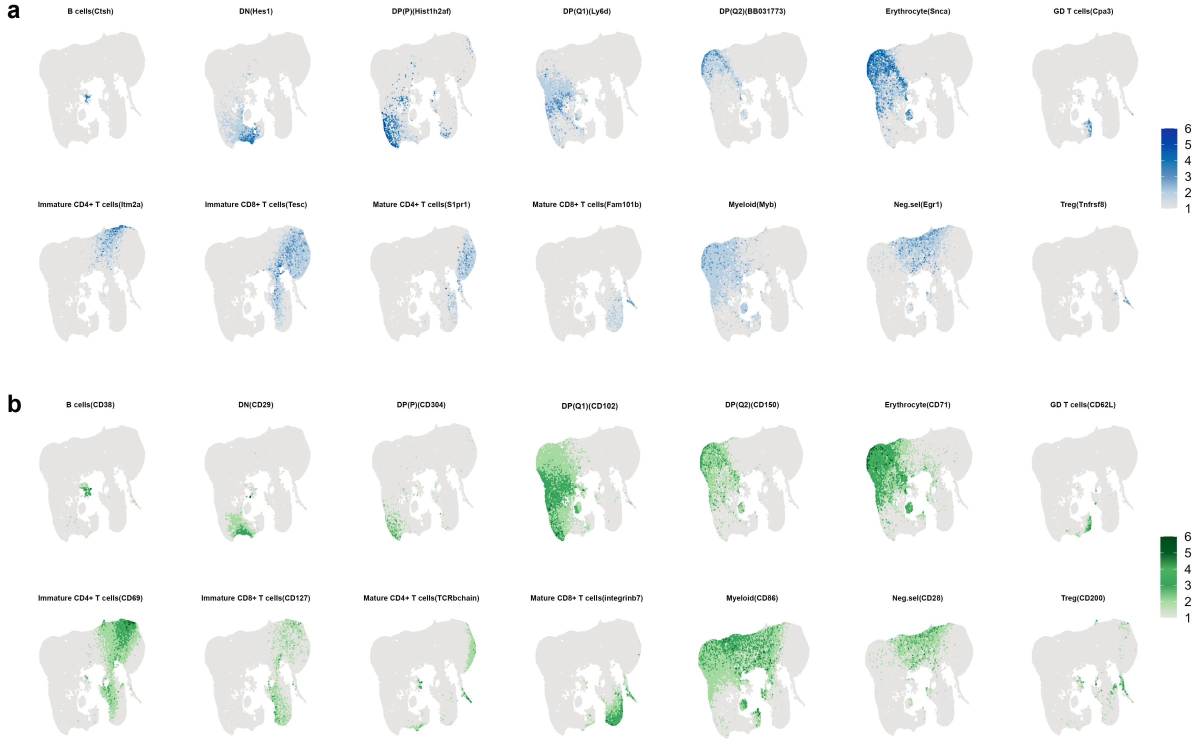

Supplementary Figure 21 Feature plot of marker RNA (a) and marker ADT (b) expression levels for each cell cluster identified by ADT-only clustering.

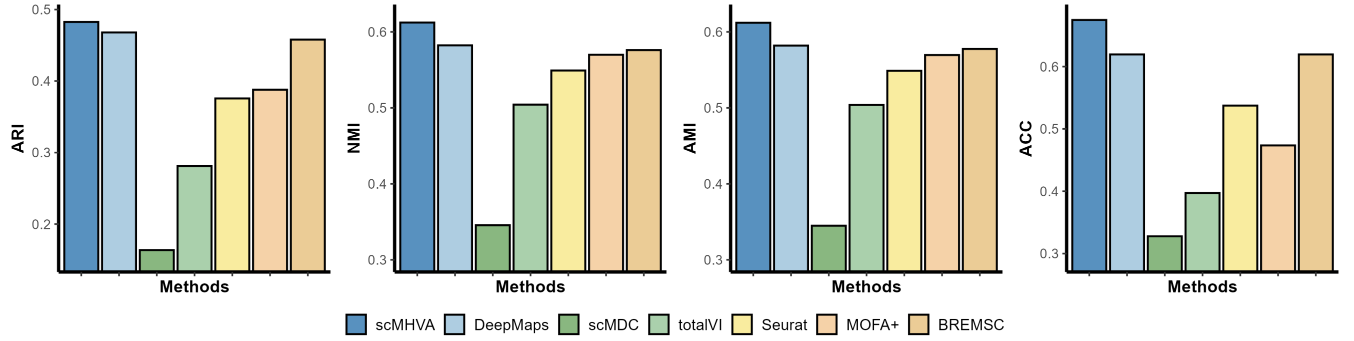

Supplementary Figure 22 Benchmarking of scMHVA in terms of cell clustering on the mouse thymocyte dataset. Comparative evaluation of clustering performance on the mouse thymocyte dataset using ARI, NMI, AMI, and ACC evaluation metrics.

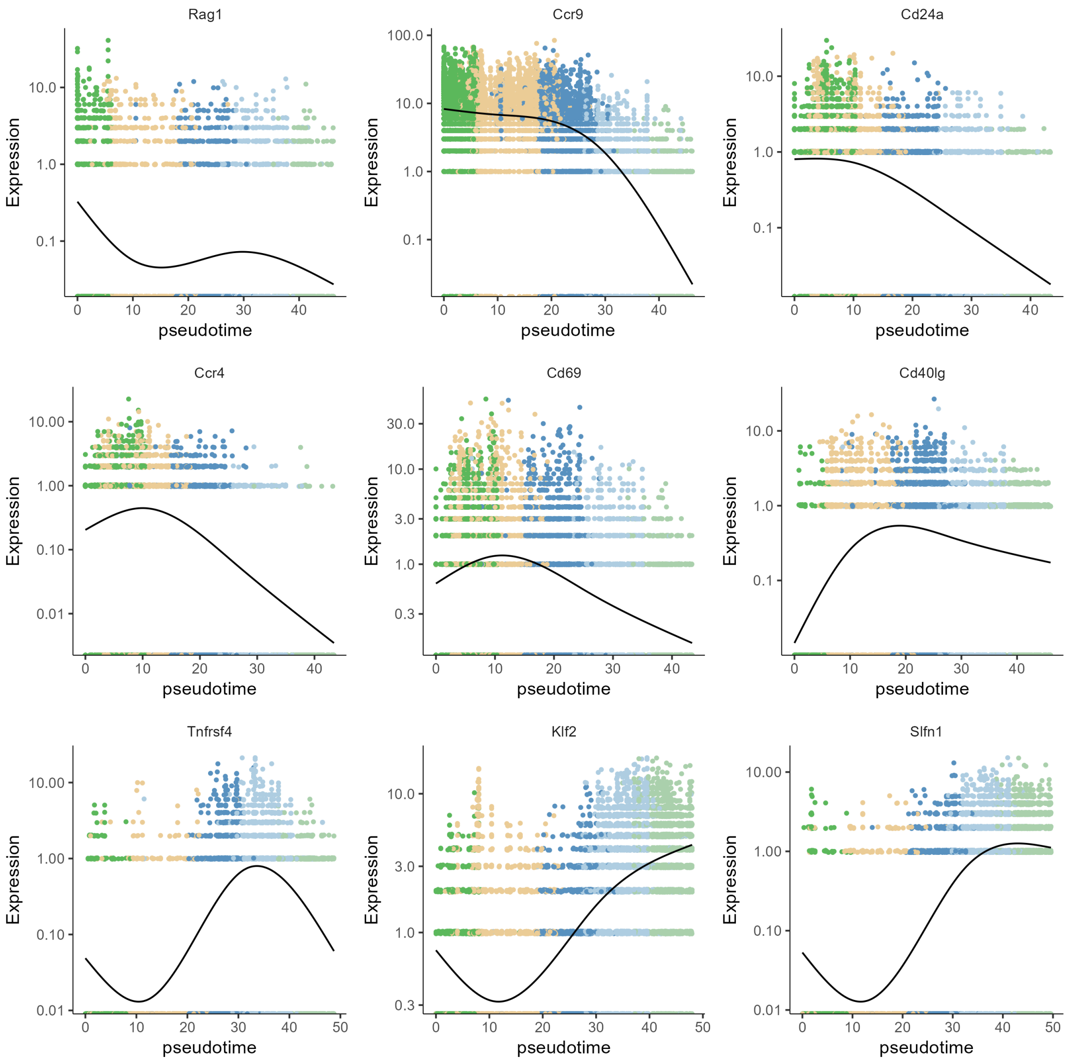

Supplementary Figure 23 Expression of features in the CD4+ T-cell lineage that vary over pseudotime.

Supplementary Note 5: Methods.

Methods	Tuned Hyperparameters	Values	Optimal Value
scMDC	gamma	0.01, 0.05,0.1,0.5,1	0.1
	sigma1	1.5, 2, 2.5, 3, 3.5	2.5
	sigma2	0.5, 1, 1.5, 2, 2.5	1.5
	phi	0.0001, 0.0005, 0.001, 0.005, 0.01	0.001
scDeepCluster	batchsize	64, 128, 256, 512	256
	gamma	0.1, 0.5, 1, 1.5	1
	sigma	1.5, 2, 2.5, 3, 3.5	2.5
scGAE	latent_dim	5, 15, 25, 35, 45	25
	W_a	0.2, 0.4, 0.6, 0.8, 1	0.4
	W_c	0.5, 1, 1.5, 2, 2.5	1.5
	W_x	0.1, 0.5, 1, 1.5, 2	1
totalVI	n_latent	10, 20, 30, 40, 50	20
	latent_distribution	normal, ln	normal
Seurat	nfeatures	1000, 1500, 2000, 2500, 3000	2000
	PCA_dim	10, 20, 30, 40, 50	30
	algorithm	1, 2, 3, 4	4
MOFA+	n_factors	5, 10, 20, 30, 40	10
BREMSC	sigmaB	0.4, 0.6, 0.8, 1, 1.2	0.8
DeepMaps	n_head	2, 4, 8, 16, 32	8
	n_hid	32, 64, 128, 256	128

Supplementary Table 4 Hyperparameter tuning details for baseline methods.

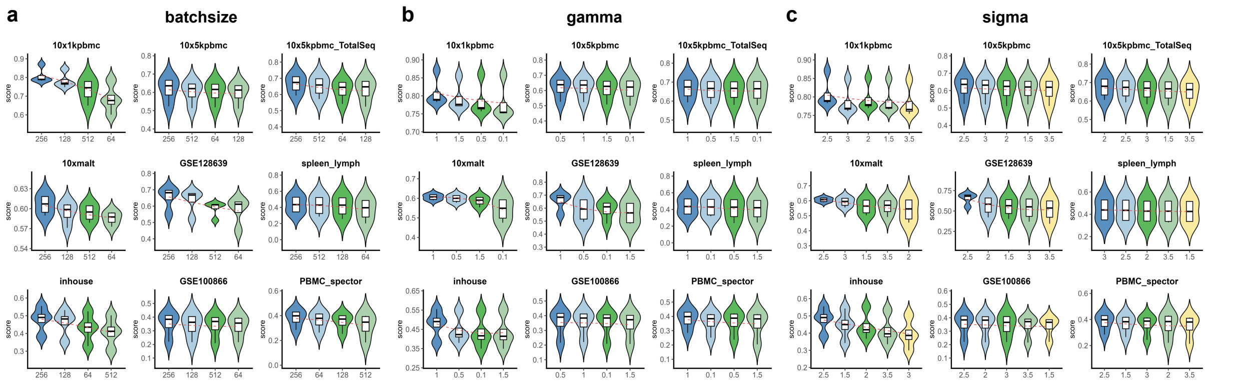

Supplementary Figure 24 Impact of different hyperparameter configurations of scDeepcluster on clustering performance across nine CITE-seq datasets.

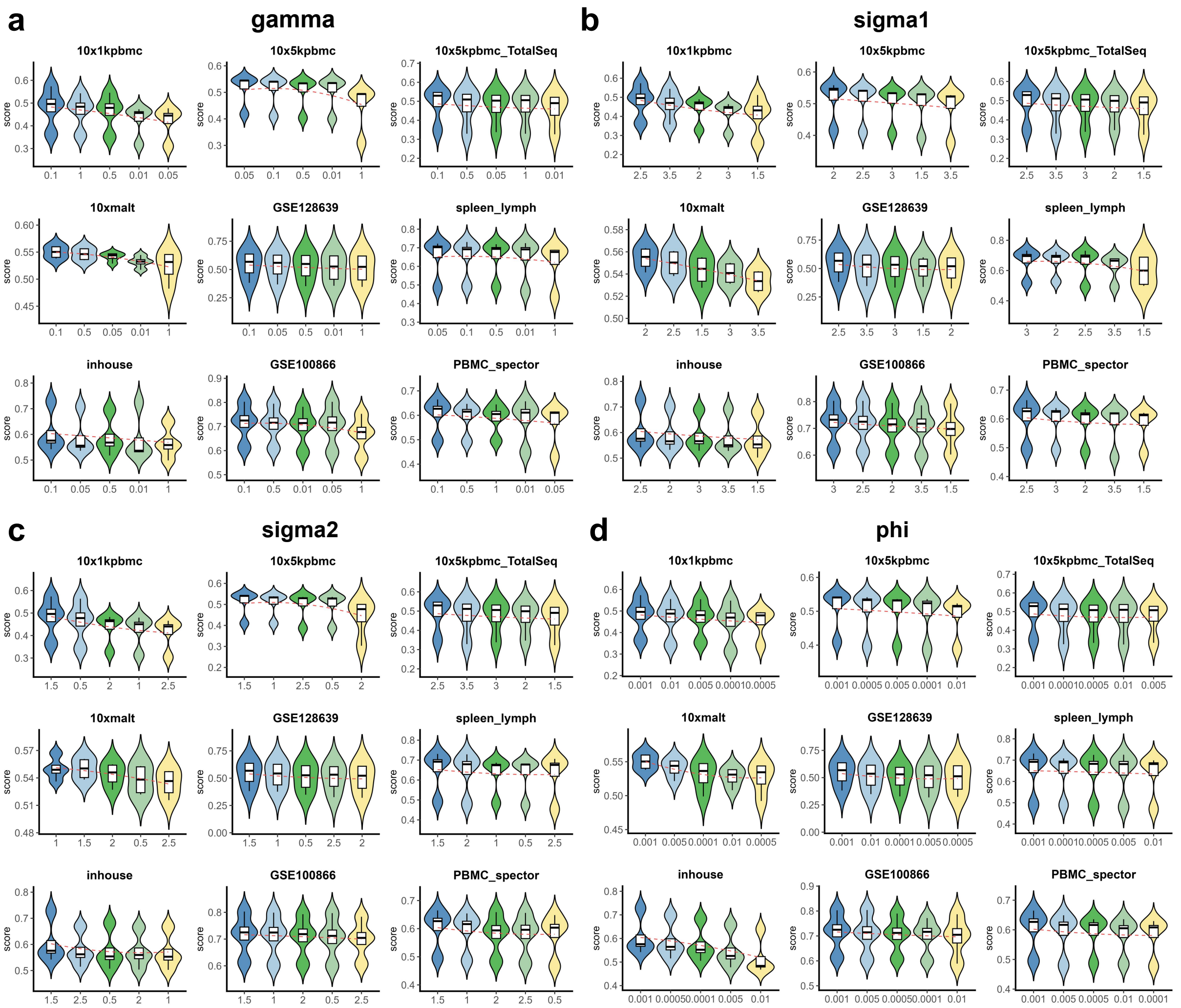

Supplementary Figure 25 Impact of different hyperparameter configurations of scMDC on clustering performance across nine CITE-seq datasets.

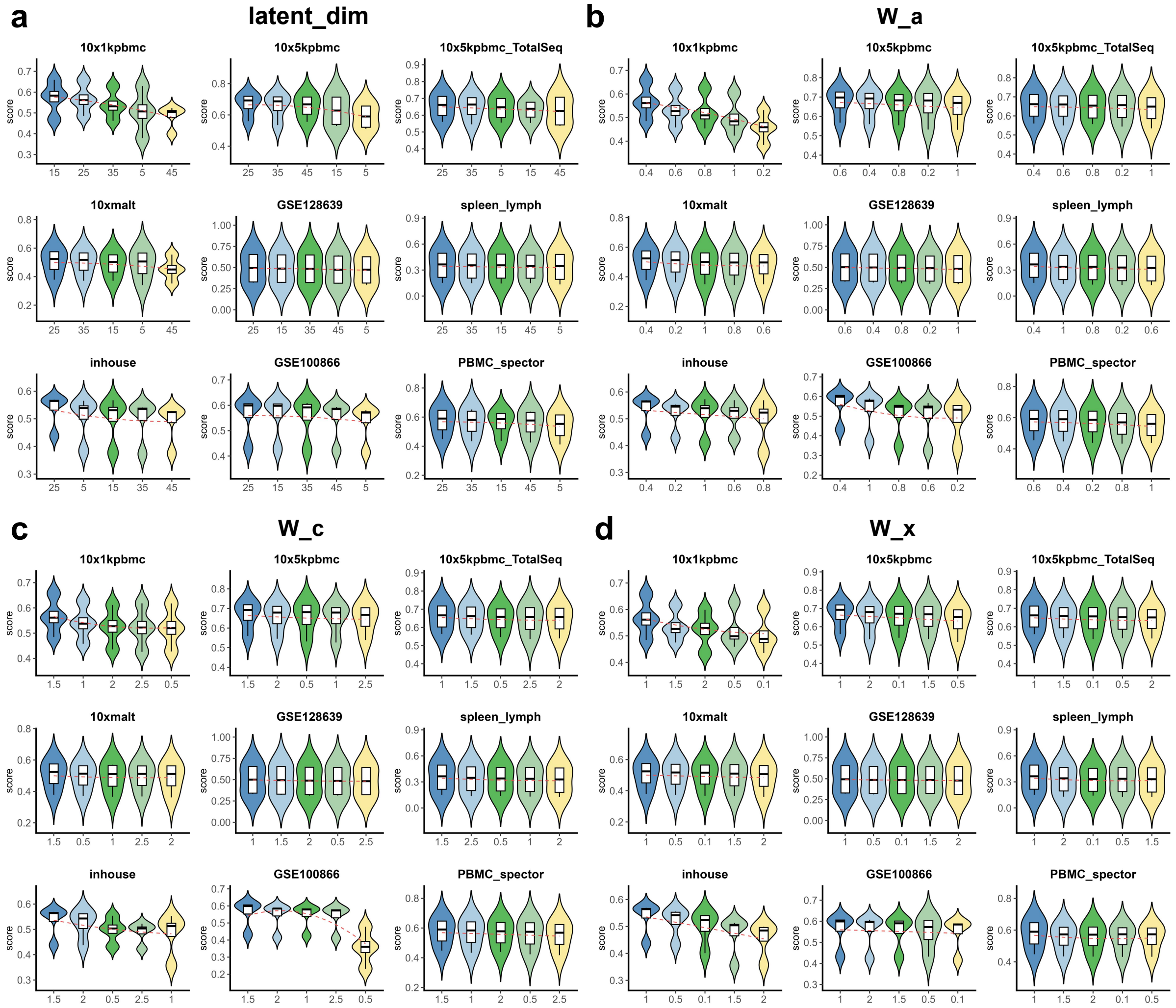

Supplementary Figure 26 Impact of different hyperparameter configurations of scGAE on clustering performance across nine CITE-seq datasets.

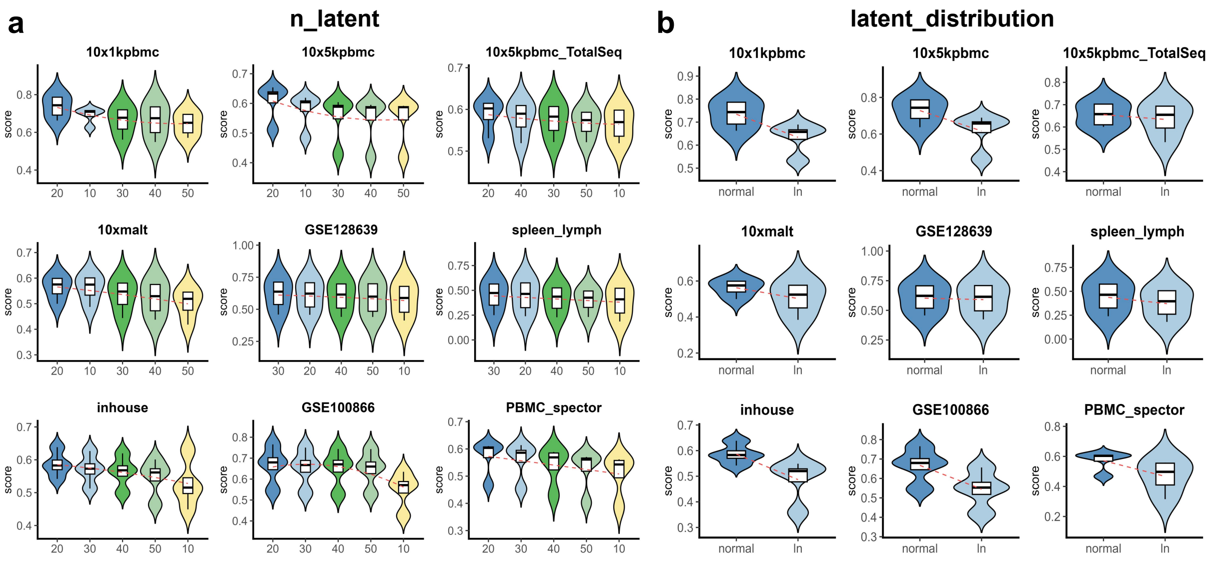

Supplementary Figure 27 Impact of different hyperparameter configurations of totalVI on clustering performance across nine CITE-seq datasets.

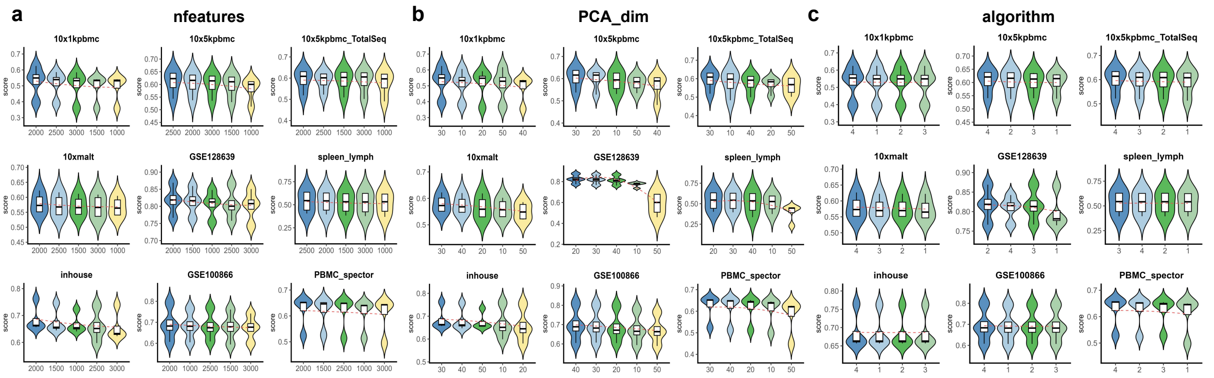

Supplementary Figure 28 Impact of different hyperparameter configurations of Seurat on clustering performance across nine CITE-seq datasets.

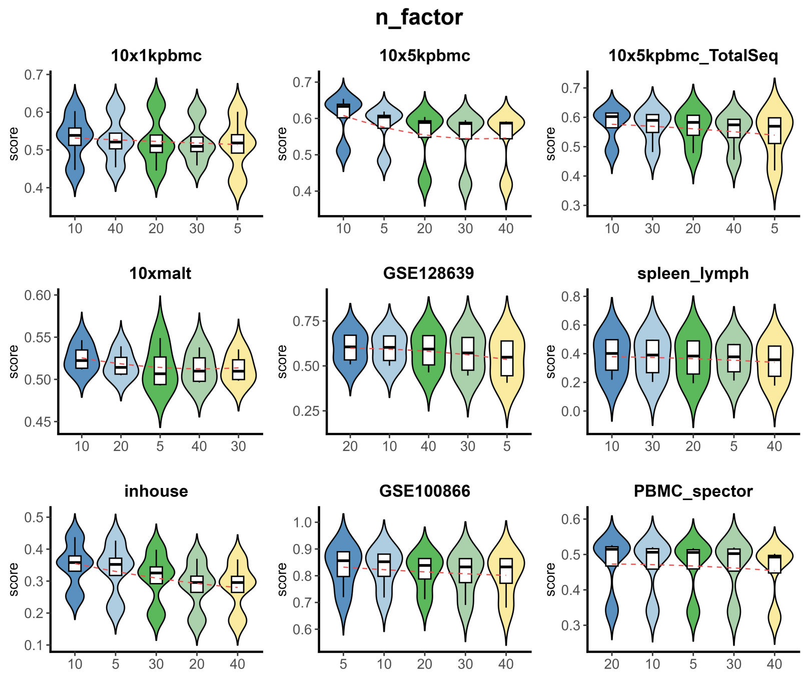

Supplementary Figure 29 Impact of different hyperparameter configurations of MOFA+ on clustering performance across nine CITE-seq datasets.

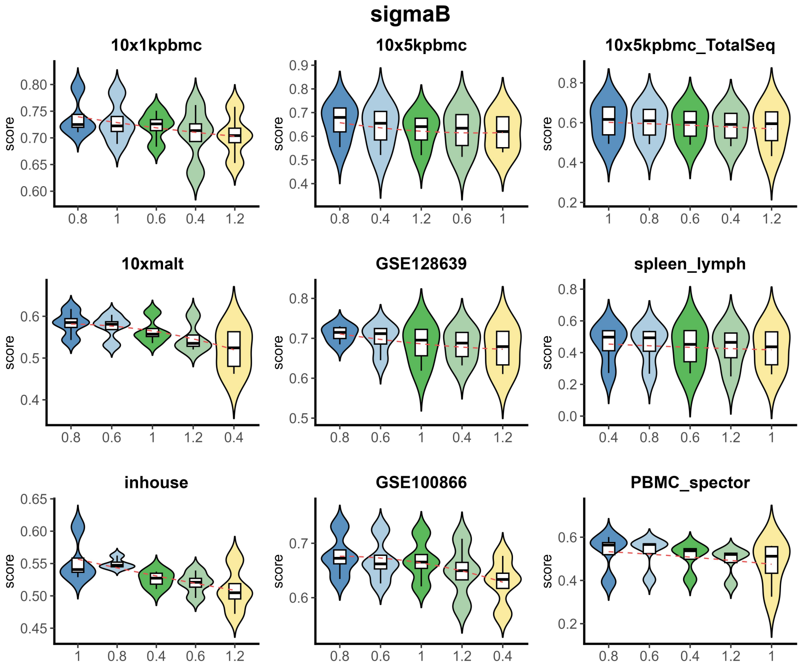

Supplementary Figure 30 Impact of different hyperparameter configurations of BREMSC on clustering performance across nine CITE-seq datasets.

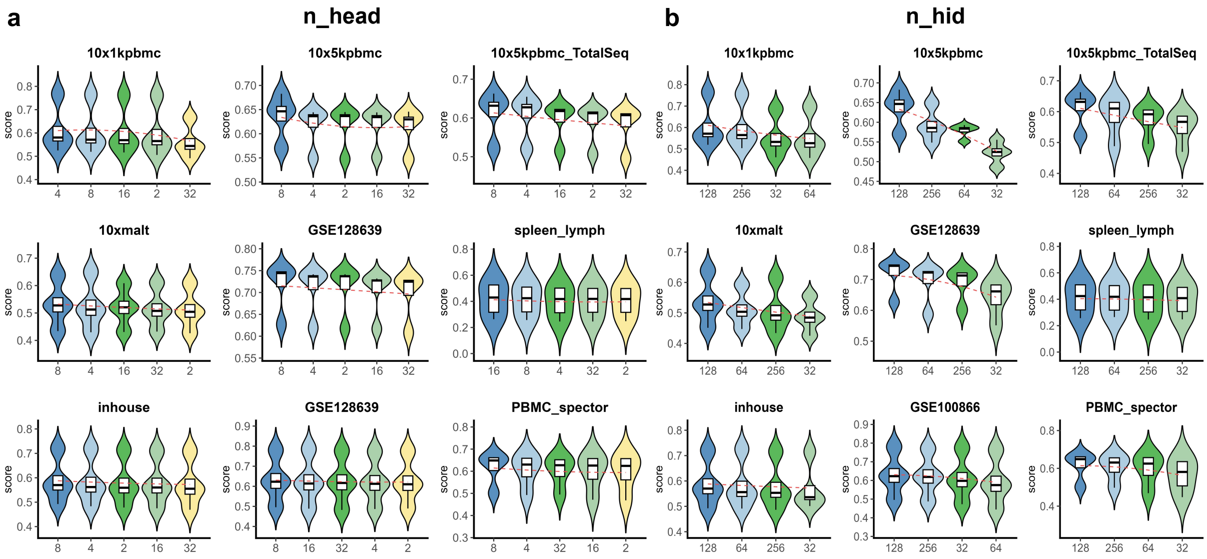

Supplementary Figure 31 Impact of different hyperparameter configurations of DeepMaps on clustering performance across nine CITE-seq datasets.

Name	Data type	Species	Sample	Cell	Gene	ADT	Class	Batch
10x1kpbmc	CITE-seq	Human	PBMCs from a healthy donor	713	33538	17	5	0
10x5kpbmc	CITE-seq	Human	PBMCs from a healthy donor	5247	33538	32	12	0
10x5kpbmc_TotalSeq	CITE-seq	Human	PBMCs from a healthy donor	5527	33538	32	12	0
10xmalt	CITE-seq	Human	Cells from a MALT Tumor	8412	33538	17	11	0
GSE100866	CITE-seq	Human	Cord blood mononuclear cells	1182	33514	10	6	0
PBMC_spector	CITE-seq	Human	Peripheral blood mononuclear cells	3762	33538	49	16	0
inhouse	CITE-seq	Human	Peripheral blood mononuclear cells	1372	33538	10	7	0
GSE128639	CITE-seq	Human	Bone marrow mononuclear cells	30672	17009	25	27	0
GSE186078	CITE-seq	Mouse	Cells from the thymus of wild-type and lineage-restricted mice	72042	5125	111	21	2
GSE194122	CITE-seq	Human	Bone marrow mononuclear cells	90261	13953	134	45	12
Haniffa COVID	CITE-seq	Human	COVID-19 patient peripheral blood immune cells and healthy controls donors	647366	24737	192	18	130

Supplementary Table 5 Details of multi-omics CITE-seq datasets.
